# Shared Genetic Basis and Causal Relationship Between Television Watching, Breakfast Skipping and Type 2 Diabetes: Evidence From a Comprehensive Genetic Analysis

**DOI:** 10.3389/fendo.2022.836023

**Published:** 2022-03-24

**Authors:** Dongze Chen, Hanyu Wu, Xinpei Wang, Tao Huang, Jinzhu Jia

**Affiliations:** ^1^ Department of Biostatistics, School of Public Health, Peking University, Beijing, China; ^2^ Department of Bioinformatics, School of Life Science, Peking University, Beijing, China; ^3^ Department of Epidemiology and Biostatistics, School of Public Health, Peking University, Beijing, China; ^4^ Key Laboratory of Molecular Cardiovascular Sciences (Peking University), Ministry of Education, Beijing, China; ^5^ Center for Statistical Science, Peking University, Beijing, China

**Keywords:** TV watching, breakfast skipping, type 2 diabetes, Mendelian randomization, genome genetic correlation

## Abstract

**Background:**

Epidemiological investigations have established unhealthy lifestyles, such as excessive leisurely sedentary behavior (especially TV/television watching) and breakfast skipping, increase the risk of type 2 diabetes (T2D), but the causal relationship is unclear. We aimed to understand how single nucleotide variants contribute to the co-occurrence of unhealthy lifestyles and T2D, thereby providing meaningful insights into disease mechanisms.

**Methods:**

Combining summary statistics from genome-wide association studies (GWAS) on TV watching (*N* = 422218), breakfast skipping (*N* = 193860) and T2D (*N* = 159208) in European pedigrees, we conducted comprehensive pairwise genetic analysis, including high-definition likelihood (HDL-method), cross-phenotype association studies (CPASSOC), GWAS-eQTL colocalization analysis and transcriptome-wide association studies (TWAS), to understand the genetic overlap between them. We also performed bidirectional two-sample Mendelian randomization (MR) analysis for causal inference using genetic instrumental variables, and two-step MR mediation analysis was used to assess any effects explained by body mass index, lipid traits and glycemic traits.

**Results:**

HDL-method showed that T2D shared a strong genetic correlation with TV watching (*r_g_* = 0.26; *P* = 1.63×10^-29^) and skipping breakfast (*r_g_* = 0.15; *P* =2.02×10^-6^). CPASSOC identifies eight independent SNPs shared between T2D and TV watching, including one novel shared locus. TWAS and CPASSOC showed that shared genes were enriched in lung, esophageal, adipose, and thyroid tissues and highlighted potential shared regulatory pathways for lipoprotein metabolism, pancreatic β-cell function, cellular senescence and multi-mediator factors. MR showed TV watching had a causal effect on T2D (β_IVW_ = 0.629, *P*_IVW_ = 1.80×10^-10^), but no significant results were observed between breakfast skipping and T2D. Mediation analysis provided evidence that body mass index, fasting glucose, hemoglobin A1c and high-density lipoprotein are potential factors that mediate the causal relationship between TV and T2D.

**Conclusions:**

Our findings provide strong evidence of shared genetics and causation between TV watching and T2D and facilitate our identification of common genetic architectures shared between them.

## Highlights

- The strongest positive genetic correlation was observed between TV watching and type 2 diabetes.
- Cross-trait meta-analysis identifies eight independent genomic loci shared between type 2 diabetes and television watching, one of which is novel.
- Implicated genes suggest potential treatment targets and signaling pathways for type 2 diabetes and television watching.
- Transcriptome-wide association studies and cross-trait meta-analysis support the role of lipoprotein metabolism, cellular senescence and multi-mediator factors may account for the shared metabolic pathway and causes between TV watching and T2D.
- Mendelian randomization study showed TV watching had strong causal effect on T2D (β_IVW_ = 0.629, *P*_IVW_ = 1.80×10^-10^).

## Introduction

Type 2 diabetes (T2D) is a global epidemic that affects more than 463 million people and is a leading cause of morbidity and mortality worldwide. Family-based studies have shown that T2D is highly heritable, with an estimated heritability range of 20%-80% (1, 2). Currently, worldwide prevalent unhealthy lifestyles (especially TV watching and breakfast skipping) are also considered to be the key contributors to T2D. However, whether such an unhealthy lifestyle is causally associated or shares a genetic basis with T2D remains largely unknown.

A growing body of evidence from observational studies suggests that the risk of T2D is positively associated with prolonged TV watching (3–6) and breakfast skipping (7–9). A prospective study showed that TV watching is always related to higher energy intake than expenditure and leads to higher BMI (10), which affects metabolism by releasing non-esterified fatty acids (NEFAs) (11). Increasing plasma NEFA levels then leads to inadequate insulin secretion and insulin resistance (low insulin sensitivity), together contributing to the development of T2D (11). The association between breakfast skipping and T2D is also reported to be partially mediated by body mass index (BMI) (9). Furthermore, breakfast skippers are more likely to have lower serum HDL cholesterol levels (12), which is widely confirmed to be associated with an increased risk of T2D in Mendelian randomization studies (13). Therefore, we hypothesized that a common genetic etiology and the mediating role of BMI or HDL may at least partially explain the association between T2D and TV watching and breakfast skipping.

Evidence from observational studies is limited for making causal inferences, as such associations may be due to (residual) confounding and/or reverse causality (14). Considering that genetics is unlikely to be influenced by these factors, it is informative to use genetic variants as instrumental variables to investigate the causal relationships behind these associations. To date, genome-wide association studies (GWAS) have been able to detect 145, 128 and 6 genome-wide significant independent SNP signals for T2D, TV watching and breakfast skipping, respectively. Many of the significant loci for TV watching are also susceptibility loci for T2D, suggesting a possible common genetic etiology between them (15–17). Meanwhile, a growing number of Mendelian randomization studies based on strong instrumental variables (IVs) have shown a causal relationship between TV watching and numerous adverse outcomes, such as cerebrovascular diseases (18), coronary artery disease (17), chronic kidney disease (19) and lung cancer (20). However, Mendelian randomization cannot deal with pleiotropy, where genetic variation is associated with multiple traits, since it will break the single pathway hypothesis of MR (21). Research suggests that cross-phenotypic (CP) associations can recognize genetic pleiotropy in human diseases and highlight shared biological pathways compared to single-trait analysis (22). However, little research has been done on CP association analysis between T2D with TV watching and breakfast skipping.

Therefore, to increase our understanding of potential causality and shared genetic architecture between TV watching, breakfast skipping and T2D, we conducted a comprehensive genetic analysis. We performed a bidirectional MR and mediation analysis using summary statistics from public external URL (https://data.mendeley.com/datasets/mxjj6czsrd/1), the Common Metabolic Diseases Knowledge Portal (CMDKP) website (for exposures) and the Diabetes Genetics Replication And Meta-analysis (DIAGRAMv3) Consortium (for type 2 diabetes). To further identify genomic loci shared between T2D and exposures, we used cross-phenotype association (CPASSOC) analysis and transcriptome-wide association (TWAS) studies to explore shared genetic components among these complex phenotypes.

## Materials and Methods

### Data Source and Study Population

The study was conducted using publicly available GWAS summary data. Details on the study characteristics, participants, and ethics declarations for each dataset can be found in the original publications (16, 17, 23). The hitherto largest GWAS of self-reported TV watching was conducted based on the United Kingdom Biobank (UKB) population cohort (*N* = 422218) (17). A total of 45.7% of participants were male, with a mean age of 57.4 [standard deviation (SD) 8.0] years at the first assessment of the cohort, and the mean daily reported leisure TV watching was 2.8 h (SD 1.5). The most recent summary results for breakfast skipping were based on a proxy-phenotype (breakfast cereal skipping) GWAS obtained from the Common Metabolic Diseases Knowledge Portal website (16), which included 193860 participants with 24-hour retrospective dietary data from the UKB. We used the T2D GWAS summary statistics from the 2017 report of the DIAGRAMv3 Consortium, consisting of 26676 T2D cases and 132532 control individuals (23). All participants were of European ancestry and had no overlap between exposure (TV watching, breakfast skipping) and outcome (T2D) samples. The location of SNPs is based on the Genome Reference Consortium Human Build 37 (GRCh37).

### Genetic Correlation Analysis

The more recent high-definition likelihood (HDL-method) (24) method and conventional cross-trait linkage disequilibrium score (LDSC) regression (25) were conducted to evaluate the genetic correlation (***r***_g_) between T2D and TV watching and breakfast skipping. HDL-method extends the LDSC method by modeling the relation between covariances among Z statistics for pairs of traits across multiple SNPs and a full matrix of cross-SNP LD scores. As the HDL-method yields more precise estimates of genetic correlations than LDSC, we chose the HDL-method as the primary result. The HDL-method uses the LD reference computed from 335265 genomic British individuals in the UKB.

### Cross Trait Meta-Analysis

Genetic correlation depicts the genome-wide average sharing of genetic effects between traits. To identify genetic variants shared between traits, we applied cross-trait GWAS meta-analysis using the cross-phenotype association (CPASSOC) (26) method to combine the association evidence for TV watching and breakfast skipping with T2D based on the criteria of both ***r***_g_ > 10% and *P*_bonferroni_ < 0.05 from HDL-method. CPASSOC combines effect estimates and standard error of GWAS summary statistics to test the hypothesis of association between a SNP and two traits and assumes that effects may exist only within a subset of traits (27). We used the heterogonous version of cross-phenotype association (SHet), which is based on a sample size-weighted, fixed-effect model and is more powerful when there is a heterogonous effect present between studies (26).

We applied PLINK1.9 clumping function (parameters: –clump-p1 2.5e-8 –clump-p2 1e-5 –clump-r2 0.4 –clump-kb 500) to determine index loci that are independent of each other, i.e., variants with P value less than 1×10^-5^ have an *r* ^2^ greater than 0.4 and less than 500 kb away from the peak will be assigned to that peak’s clump. We identified all genes falling within each clump region. A P value of 2.5×10^-8^ (5×10^-8^/2) was used as genome-wide significance level for cross-trait meta-analysis to account for 2 meta-analyses. SNPs with a meta-analysis *P* value less than 2.5×10^-8^ and trait-specific *P* value less than 1×10^-5^ were selected for downstream analysis.

### GWAS-eQTL Coloclization Analysis

To investigate whether the shared index SNPs from CPASSOC and their expression quantitative trait loci (eQTLs) co-localized with candidate causal variants, we performed colocalization analysis, COLOC, which uses Bayesian posterior probability to assess colocalization (28). We extracted cis-eQTL data from the Genotype-Tissue Expression (GTEx) Portal v7 for 48 single tissues (29). The SNP-associated locus was defined as within a 1-Mb window for each of the shared SNPs. The posterior probability H4 hypothesis was calculated to determine whether shared SNPs are associated with two traits. In our study, loci with posterior probability H4 > 0.9 were considered to be co-localized.

### Transcriptome-Wide Association Studies

For TV watching, skipping breakfast and T2D, we used transcriptome-wide association studies (TWAS) to identify genes whose cis-regulated gene expression was associated with the corresponding traits. Then, we further evaluated shared tissue-gene pairs between different traits. We performed TWAS analysis using FUSION software and its precomputed transcript expression reference weights, as well as eQTL data from GTEx v.7 (30). Bonferroni correction was applied to determine significant association results after multiple comparisons for all tissue-gene pairs tested for each trait (***P***_Bonferroni_ < 0.05). To increase the significance of the TWAS results, we used the most recent and authoritative summary data for T2D obtained from DIAGRAM. This study was performed in 2018 by Mahajan et al., who mined additional novel T2D susceptibility SNP loci by combining data from 898130 (including UKB sample) individuals of European descent (31).

### Mendelian Randomization Analysis

Finally, we implemented a bidirectional MR using TwoSample MR package to test the causal relationship between T2D and unhealthy lifestyles, where the associations for IV-exposure and IV-outcome came from two nonoverlapping groups of participants. Since different MR methods have different degrees of explanation and contexts of application and differ in statistical efficiency, we adopt many MR methods to estimate causal effects. The causal effect estimates from the multiplicative random effects inverse variance weighted (IVW) model were used as the primary result. We conducted a range of sensitivity analyses using multiplicative random effects inverse variance weighted heterogeneity test, weighted median, MR–Egger regression, MR-Steiger, MR-Robust Adjusted Profile Scores (MR-RAPS), MR-Pleiotropy Residual Sum and Outlier (MR-PRESSO) analysis and leave-one-out cross-validation analysis. The weighted median approach provides consistent and robust estimates even if more than 50% of the IVs are invalid (32). The intercept of MR–Egger regression can be used to evaluate the directional pleiotropy of IVs (33). We applied MR-Steiger to assure that the causal direction between the hypothesized exposure and outcome was correctly assigned (34). Considering the measurement error in SNP exposure effects, MR-RAPS is unbiased when there are many weak instruments and is robust to systematic and idiosyncratic pleiotropy (35). MR-PRESSO and leave-one-out cross-validation analysis are mainly used to detect anomalous IVs (36, 37).

Furthermore, the effect allele frequency reported in the corresponding GWAS was used to detect and exclude all palindromic SNPs to determine the corresponding strand between two GWAS in harmonization section. For trait pairs with significant causal relationships, we searched the GWAS catalog (https://www.ebi.ac.uk/gwas/) to exclude IVs with genome-wide significance for potential confounding traits (e.g., educational attainment, cognitive performance, smoking behavior, alcohol consumption, hypertension, BMI, waist-to-hip ratio, body fat percentage, cardiovascular disease, etc.) and reran the MR to obtain more robust MR estimates. For TV watching, breakfast skipping and T2D, independent genetic instruments were selected at GWAS p value < 5×10^-8^ and LD *r* ^2^ < 0.001 based on the 1000 Genomes European phase 3 reference panel. Given the multiple comparisons, in this study, we considered a *P* threshold < 0.05 as suggestive significance, while Bonferroni-corrected *P* threshold was used as statistically significant (*P*<0.05/6 = 0.008).

**Figure 1:**
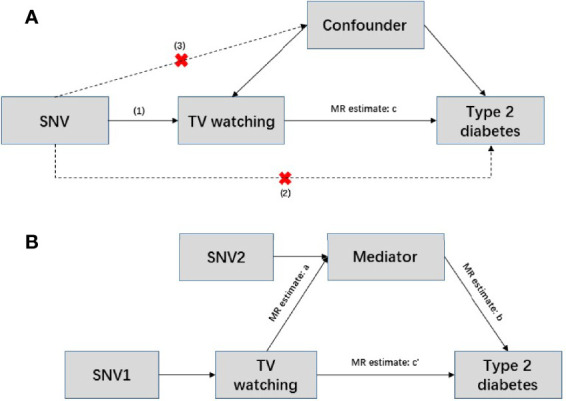
Conceptual diagram of Mendelian randomization and mediation analysis. **(A)** Mendelian randomization is based on the following three assumptions. (1) Genetic variants are strongly associated with exposure (p<5×10^-8^); (2) instrumental variables can only act on the outcome through exposure, and there is no direct association with the outcome; and (3) instrumental variables are independent of any confounding factors. In this situation, c represents the total effect, SNV: single nucleotide variant. **(B)** Two-step Mendelian randomization, where a represents the effect of the exposure on the mediator; b represents the effect of mediator on the outcome; c’ represents the direct effect; and a and b are estimated separately using separate genetic instrumental variables for both the exposure and mediator. These estimates are then multiplied together to estimate the indirect effect of the mediator (a * b), and the direct effect c’ = c – a*b.

To further assess the direct effects of TV watching on T2D, we performed two-step MR mediation analysis. We selected body mass index (BMI), 4 lipid traits [including high density lipoprotein (HDL) cholesterol, low density lipoprotein (LDL) cholesterol, triglyceride (TG), total cholesterol (TC)], and 6 glycemic traits [including fasting glucose (FG), fasting insulin (FI), 2-h postprandial glucose (2hGlu), hemoglobin A1c (HbA1c), homeostatic model assessment of beta cell function (HOMA-β), homeostatic model assessment of insulin resistance (HOMA-IR)] as potential mediators of liability to TV watching in T2D. Two-step MR is based on the coefficient product method to calculate indirect (or mediator) effects (**Figure 1**). This process involves calculating two MR estimates, one for the causal effect of exposure on the mediator and the other for the causal effect of the mediator on the outcome. These two estimates are then multiplied together to estimate the indirect effect (38). GWAS summary statistics for BMI, lipid traits, and glycemic traits were obtained from the Genetics of ANthropometric Traits (GIANT) Consortium, the Meta-Analysis of Glucose and Insulin-related traits Consortium (MAGIC), and the Global Lipid Genetics Consortium (GLGC), respectively. The source literature corresponding to the three mediated traits can be found here (39–42). There was no sample size overlap between exposures and mediators and little overlap between mediators and outcomes in the selected GWAS data. Bonferroni-corrected *P* threshold (*P*<0.05/11) was used as statistical significance accounting for the 11 mediation analyses.

## Results

### Genetic Correlations

T2D showed a strong positive genetic association with TV watching (***r***_g_ = 0.26; *P* = 1.63×10^-29^) and skipping breakfast (***r***_g_ = 0.15; *P* =2.02×10^-6^). The results suggested a potential common genetic basis and thus warranted further investigation of the underlying mechanisms using cross trait meta-analysis and instrumental variable analysis (**Table 1**).

**Table 1:** Genetic correlation of type 2 diabetes with TV watching and breakfast skipping, estimated by high-definition likelihood method (HDL-method) and linkage disequilibrium score regression (LDSC).

Method	Trait	*r*_g_	SE	*r*_g_, 95%CI	pvalue	h^2(SE)
**HDL-method**	TV watching	0.26	0.023	0.21 to 0.31	1.63E-29	0.13(0.004)
	breakfast skipping	0.15	0.032	0.09 to 0.21	2.02E-6	0.05(0.002)
**LDSC**	TV watching	0.28	0.030	0.22 to 0.34	1.28E-21	0.13(0.004)
	breakfast skipping	0.14	0.043	0.06 to 0.22	1.30E-3	0.05(0.003)

Summary statistics for each trait were merged with Hapmap3 SNPs excluding the HLA region to estimate r_g_; p value < 0.05/2;

h^2 indicates the heritability of the corresponding phenotype.

### Cross Trait Meta-Analysis

We identified eight index loci shared between T2D and TV watching (*P*_meta_ < 2.5×10^-8^ and single-trait *P* < 1×10^-5^). However, we did not find any shared loci between T2D and breakfast skipping. GWAS-eQTL colocalization analysis had no significant results, but it identified a specific region at 12q14.3 that might be an expression quantitative trait locus between T2D and TV watching (tissue: lung, mapped gene: *HMGA2*, *P*_nominal_ = 1.79×10^-4^, H4 = 1.29×10^-3^). Two of our CPASSOC index SNPs are located at the 12q14.3 region mapping to *HMGA2* gene. *HMGA2* encodes a protein belonging to the non-histone chromosomal high-mobility group (HMG) protein family, and the protein contains structural DNA-binding domains and may act as a transcriptional regulating factor. Significantly higher expression of *HMGA2* mRNA in white adipose tissue has been reported in patients with T2D (43).

More importantly, we identified one novel locus shared between T2D and TV watching (11q13.1, index SNP: rs78028320, mapped gene: *CFL1*, *P*_meta_ = 2.68×10^-9^). *CFL1* is a typical protein-coding gene that encodes cofilin-1, an intracellular actin regulatory protein that plays an important role in regulating the organization of the actin cytoskeleton. Phosphorylated (inactive) cofilin-1 is upregulated in diabetic glomeruli, suggesting alterations in actin dynamics (44). In addition, podocytes in glomeruli are the key structure for maintaining the selective filtration barrier of the kidney. Its loss and structural abnormalities contribute to the progression of diabetic nephropathy (45). It has also been reported that mice deleted of *CFL1* in podocytes developed increased albuminuria and developed renal dysfunction, as indicated by a rise in creatinine (46).

The most significant locus overall was index SNP rs4420638 (mapped gene: *APOC1*, *P*_meta_ = 2.42×10^-14^). The mapped gene *APOC1* (apolipoprotein C1) is a protein-coding gene engaged in the inhibition of cholesteryl ester transfer protein (CETP). A study showed that *APOC1* was highly expressed in clear cell renal cell carcinoma (47), and a variant of *APOC1* called T45S led to elevated rates of T2D (48). The second strongest SNP was rs4565329 (mapped gene: *CENPW*, *P*_meta_ = 7.64×10^-14^). *CENPW* encodes a centromere protein that plays a central role in the assembly of kinetochore proteins, mitotic progression and chromosome segregation. The association between *CENPW* and T2D has been reported in previous genome-wide meta-analysis (49). SNP rs74333814 was also shared between TV watching and T2D (mapped gene: *ARAP1*, *P*_meta_ = 3.84×10^-13^). *ARAP1* encodes protein that is thought to regulate the cell-specific trafficking of a receptor protein involved in apoptosis. Findings suggest that *ARAP1* engages in islet insulin content and secretion and is thus likely to mediate the effects on diabetes susceptibility (50). Significantly, previous studies also showed that *APOC1* (51) and *ARAP1* (52) had a significant effect on BMI.

### Transcriptome-Wide Association Studies

**Figure 2:**
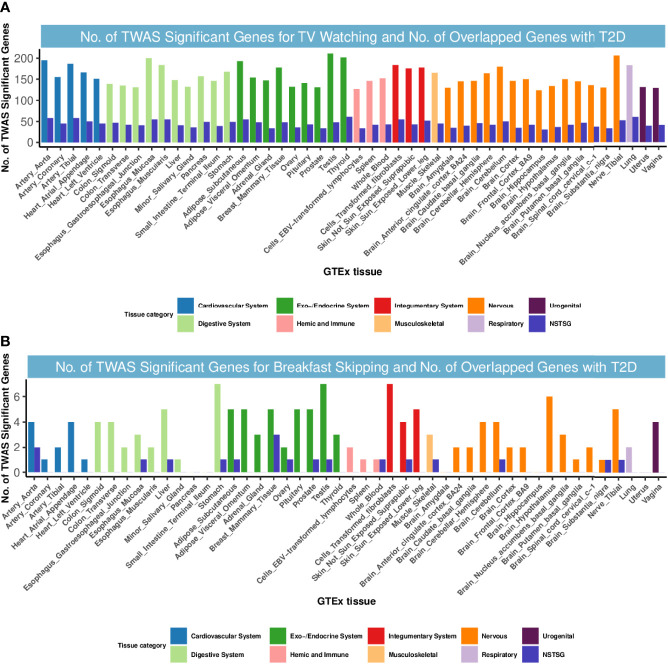
Numbers of significant genes related to TV watching and breakfast skipping and the number of shared genes with T2D. Significant genes were identified by *P*_Bonferroni_ < 0.05. GTEx, genotype-tissue expression project; GWAS, genome-wide association studies; TWAS, transcriptome-wide association study; NSTSG, Number of shared TWAS significant genes between traits; T2D: type 2 diabetes. **(A)** No. of TWAS Significant Genes for TV watching and No. of Overlapped Genes with T2D. **(B)** No. of TWAS Significant Genes for breakfast skipping and No. of Overlapped Genes with T2D.

We next delved into the genetic level and examined shared TWAS genes between TV watching, breakfast skipping and T2D. After Bonferroni correction, a total of 10127 gene-tissue pairs were found to be significantly associated with T2D in 48 GTEx tissues, in addition to 7540 and 143 gene-tissue pairs associated with TV watching and breakfast skipping, respectively. We found 365 TWAS-significant genes shared between T2D and TV watching, with significant system-wide overlap, especially in the endocrine system, cardiovascular system, digestive system and nervous system (**Figure 2**). Intriguingly, 6 of the 365 shared TWAS-significant genes were also identified in CPASSOC, including *CENPW*, *ARAP1*, *CFL1*, *HMGA2*, *ABO* and *ATG16L2*. The functions of the first four genes have been described in detail in the CPASSOC section, and here, we focus on the two genes *ABO* and *ATG16L2*. The *ABO* (9q34.2) gene encodes the blood group ABO systemic transferase and is ubiquitously expressed in many tissues and cell types (53). Genetic variation at the ABO locus and ABO blood group have been found to be associated with the risk of venous thromboembolism (54) and type 2 diabetes (55). *ATG16L2* (11q13.4) is a protein-coding gene whose function is not fully understood, and it has been shown to play a unique function in autophagy. Analysis of transcriptomic data shows that autophagy plays a major role in the molecular pathology of T2D and AD (56).

However, for T2D and breakfast skipping, we observed only 12 shared TWAS-significant genes, mainly enriched in the endocrine system (**Figure 2**). Notably, we found that *EIF2S2P3* was the most enriched and significant among the 12 shared genes. *EIF2S2P3* is located at 10p23.33 and is a pseudogene. It has been reported to be associated with T2D (56), but its function remains unclear.

### Mendelian Randomization Analysis

**Figure 3:**
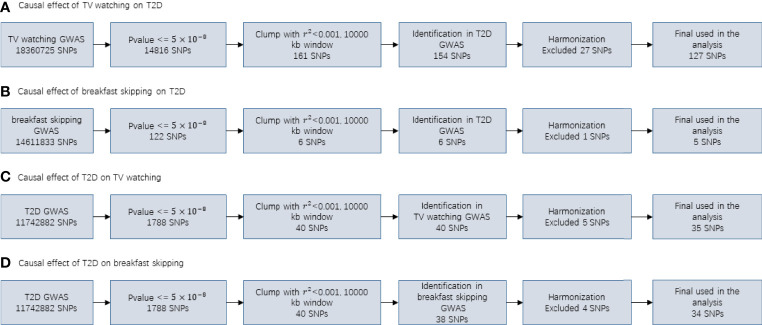
Flowcharts visualizing the process for instrument definition, extraction and harmonization for the two-sample MR analyses conducted in the present study.

In our MR study, for T2D, TV watching, and breakfast skipping, we selected 35, 127 and 5 SNPs as IVs, respectively. The detailed characteristics of the IVs are shown in **Tables S1**-**S4**, and the screening flow of IVs is shown in **Figure 3**. F statistics provide an indication of the strength of the instrument and can be calculated using formula $F=\frac{n-k-1}{k}\cdot \frac{r^{2}}{1-r^{2}}$ (*n* is sample size, *k* is the number of IVs, and *r* ^2^ refers to how much variation in the trait can be explained by the set of genetic instruments used) (57). Given that *r* ^2^ is not generally provided in GWAS summary data, we used the formula $r^{2}=\sum^{}\left[\frac{\beta^{2}\cdot 2\cdot f\cdot \left(1-f\right)}{\beta^{2}\cdot 2\cdot f\cdot \left(1-f\right)+se^{2}\cdot 2\cdot n\cdot f\cdot \left(1-f\right)}\right]$ (*f* is effect allele frequency, *n* is sample size, *β* is effect estimate for each SNP and *se* is standard error for each SNP) (58) to obtain *r* ^2^ estimates. The *F* statistics for T2D, TV watching and breakfast skipping IVs are 69.86, 142.42 and 49.69, respectively (*F >*10 demonstrates that the analysis is unlikely to be affected by weak instrumental bias) (59).

As shown in **Table 2**, TV watching was positively associated with the risk of type 2 diabetes [OR (95% CI)_IVW_ = 1.86 (1.54, 2.26), *P* = 1.80×10^-10^; OR_WM_ = 1.82 (1.43, 2.32), *P* = 1.12×10^-6^; OR_MR-RAPS_ = 1.78 (1.50, 2.11), *P* = 3.13×10^-11^; OR _MR-PRESSO : Outlier-corrected_ = 1.84 (1.56, 2.16), *P* = 1.22×10^-11^], with all P values reaching the Bonferroni-corrected threshold and without any evidence of pleiotropy (*P*_MR-Egger-intercept_ = 0.41). This causal effect became more significant in the sensitivity analysis excluding 16 SNPs associated with potential confounders (**Table 3**) [OR (95% CI)_IVW_ = 1.94 (1.60, 2.36), *P* = 3.74×10^-11^; OR_WM_ = 1.82 (1.41, 2.35), *P* = 3.27×10^-6^; OR_MR-RAPS_ = 1.78 (1.50, 2.11), *P* = 3.13×10^-11^; OR _MR-PRESSO : Outlier-corrected_ = 1.84 (1.56, 2.16), *P* = 1.22×10^-11^]. The confounding traits associated with the 16 SNPs can be found in **Table S5**. However, there was no significant causal effect estimate from breakfast skipping to T2D. Due to shared biological pathways, T2D may further influence unhealthy lifestyles. To explore whether there is reverse causality, we performed an inverse MR analysis. We did not observe any significant association between genetic predisposition to T2D with TV watching and breakfast skipping (**Table 2** all *P* > 0.05). The leave-one-out cross-validation analysis showed that the overall estimates were not overdriven by any particular SNP (**Figures S1**-**S4**). The MR Steiger results showed that all causal estimates were in the intended direction (all ***P***_MR Steiger_ ≪ 0.05, **Table 2**). The nearly symmetric funnel plots indicate no evidence of pleiotropy in the analysis (**Figures S5**-**S8**). In summary, instrumental variable analysis suggests a potential causal effect of increased TV watching time on an increased risk of T2D.

**Table 2:** Causal relationships between TV watching, skipping breakfast and T2D (findings adjusted for multiple comparisons).

Exposure	Outcome	N_snp	Method	beta	OR	95%CI^#^	SE	p_value	Heterogeneity_P_value	Intercept_P_value	Steiger_P_value
**TV watching**	T2D	127	IVW	0.629	1.86	(1.54,2.26)	0.098	**1.80E-10**	1.66E-05	NA	1.12E-168
			WM	0.599	1.82	(1.44,2.3)	0.12	**6.36E-07**	NA	NA	
			MR–Egger	0.253	1.29	(0.52,3.17)	0.46	5.83E-01	1.60E-05	0.41	
			MR-RAPS	0.577	1.78	(1.5,2.11)	0.087	**3.13E-11**	NA	NA	
			MR-PRESSO:raw	0.569	1.77	(1.49,2.09)	0.086	**6.07E-10**	NA	NA	
			MR-PRESSO : Outlier-corrected	0.609	1.84	(1.56,2.16)	0.083	**1.22E-11**	NA	NA	
**skipping breakfast**	T2D	5	IVW	0.232	1.26	(0.51,3.14)	0.465	6.18E-01	0.11	NA	3.67E-16
			WM	0.752	2.12	(0.89,5.07)	0.444	8.99E-02	NA	NA	
			MR–Egger	2.111	8.25	(0.31,219.57)	1.674	2.97E-01	0.16	0.33	
			MR-RAPS	0.255	1.29	(0.63,2.67)	0.37	4.90E-01	NA	NA	
			MR-PRESSO:raw	0.239	1.27	(0.6,2.69)	0.383	5.61E-01	NA	NA	
			MR-PRESSO: Outlier-corrected	NA	NA	NA	NA	NA	NA	NA	
**T2D**	TV watching	35	IVW	-0.003	NA	(-0.017 0.011)	0.007	6.16E-01	3.04E-09	NA	2.87E-290
			WM	0.001	NA	(-0.011,0.013)	0.006	8.79E-01	NA	NA	
			MR–Egger	0.012	NA	(-0.021,0.045)	0.017	4.82E-01	4.78E-09	0.34	
			MR-RAPS	-0.002	NA	(-0.016,0.012)	0.007	7.55E-01	NA	NA	
			MR-PRESSO:raw	-0.002	NA	(-0.014, 0.010)	0.006	8.07E-01	NA	NA	
			MR-PRESSO: Outlier-corrected	-0.001	NA	(-0.011, 0.009)	0.005	8.85E-01	NA	NA	
**T2D**	skipping breakfast	34	IVW	-0.002	NA	(-0.016, 0.012)	0.007	7.72E-01	1.29E-03	NA	1.28E-203
			WM	-0.001	NA	(-0.017, 0.015)	0.008	9.25E-01	NA	NA	
			MR–Egger	0.009	NA	(-0.024, 0.042)	0.017	5.99E-01	1.15E-03	0.49	
			MR-RAPS	0.004	NA	(-0.010, 0.018)	0.007	5.24E-01	NA	NA	
			MR-PRESSO:raw	0.002	NA	(-0.010,0.014)	0.006	7.58E-01	NA	NA	
			MR-PRESSO : Outlier-corrected	-0.001	NA	(-0.013,0.011)	0.006	9.08E-01	NA	NA	

T2D, type 2 diabetes; CI, confidence interval; IVW, inverse variance weighted; MR, Mendelian randomization; NA, not applicable; N_snp: number of instrumental variables; OR, odds ratio; SE, standard error; SNP, single nucleotide polymorphism; WM, weighted median. When T2D is used as the outcome, there is an OR value.

: 95% CIs of ORs are presented for the analysis of T2D as outcome, while 95% CIs of β values are presented for the analysis of the other outcomes.

p_value in bold refers to achieving statistical significance (p_value < 0.05/6).

**Table 3:** The association between TV watching and risk of type 2 diabetes after remove 16 SNPs associated with confounding traits.

Exposure	Outcome	N_snp	Method	beta	OR	CI	SE	p_value	Heterogeneity_P_value	Intercept_P_value	Steiger_P_value
**TV watching**	**T2D**	111	IVW	0.66	1.94	(1.6,2.36)	0.1	3.74E-11	1.66E-05	NA	1.1E-168
		111	WM	0.59	1.82	(1.41,2.35)	0.129	3.27E-06	NA	NA	1.1E-168
		111	MR Egger	0.60	1.83	(0.71,4.69)	0.481	0.21	1.60E-05	0.41	1.1E-168
		111	MR-RAPS	0.58	1.78	(1.5,2.11)	0.087	3.13E-11	NA	NA	1.1E-168
		111	MR-PRESSO:raw	0.57	1.77	(1.49,2.09)	0.086	6.07E-10	NA	NA	1.1E-168
		111	MR-PRESSO: Outlier-corrected	0.61	1.84	(1.56,2.16)	0.083	1.22E-11	NA	NA	1.1E-168

T2D, type 2 diabetes; CI, confidence interval; IVW, inverse variance weighted; MR, Mendelian randomization; NA, not applicable; N_snp, number of instrumental variables; OR, odds ratio; SE, standard error; SNP, single nucleotide polymorphism; WM, weighted median. When T2D is used as the outcome, there is an OR value.

Epidemiological studies have shown that prolonged TV watching leads to increased BMI (60), lower HDL cholesterol (61), and higher fasting glucose concentrations (62) and that BMI and blood glycolipid traits are known risk factors for T2D (63), suggesting a potential mediating role for these traits in the association between TV watching and T2D. We performed a two-step MR mediation analysis to explain the mediation proportion for BMI, 4 lipid traits, and 6 glycemic traits. As shown in **Table 4**, the results revealed that four potential mediators produced a significant mediating effect. After adjusting for HbA1c, FG, and HDL, the estimates of causal effects produced moderate attenuation (OR: 1.78 adjusted for HbA1c, 1.71 adjusted for FG and 1.75 adjusted for HDL). In contrast, the association between TV watching and the risk of T2D was much more attenuated after adjusting for BMI (OR: 1.55 adjusted for BMI). Mediation analysis showed that the causal association between TV watching and T2D risk was partially mediated by BMI (mediation percentage = 29.10%), FG (mediation percentage = 13.51%), HDL (mediation percentage = 9.86%) or HbA1c (mediation percentage = 7.31%). Adjusting for these four factors simultaneously and adjusting for each factor separately produced results that were in the same direction as the results without adjustment, although the effect size was attenuated. In addition, we did not observe significant mediating effects for the other 7 glycemic-lipid traits.

**Table 4:** Two-step Mendelian randomization mediation analysis of the association between TV watching (exposure) and type 2 diabetes (outcome).

Mediator	Exposure → Mediator			Mediator → Outcome			Indirect causal effect by coefficient product	Direct causal effect	Adjust OR	Proportion of mediation
	IVW causal effect	IVW p value	MR Egger Intercept p value	IVW causal effect	IVW p value	MR Egger Intercept p value				
**Adjust for BMI**	0.315	**2.76E-06**	0.195	0.581	**5.14E-04**	0.563	0.183	0.439	1.55	29.10%
**Adjust for TC**	0.112	1.08E-01	0.119	-0.1	4.21E-02	0.307	NA	NA	NA	NA
**Adjust for TG**	0.24	**3.18E-06**	0.207	0.106	1.61E-01	0.028	NA	NA	NA	NA
**Adjust for HDL**	-0.289	**1.22E-05**	0.002	-0.213	**7.15E-04**	0.008	0.062	0.561	1.75	9.86%
**Adjust for LDL**	0.171	6.58E-03	0.197	-0.033	4.96E-01	0.344	NA	NA	NA	NA
**Adjust for FG**	0.053	**9.45E-04**	0.589	1.602	**4.03E-08**	0.015	0.085	0.537	1.71	13.51%
**Adjust for FI**	0.088	**1.09E-06**	0.898	1.318	6.19E-02	0.253	NA	NA	NA	NA
**Adjust for HOMA-β**	0.074	**4.01E-03**	0.862	-2.595	1.76E-01	0.221	NA	NA	NA	NA
**Adjust for HOMA-IR**	0.176	**2.13E-08**	0.596	0.346	2.03E-01	0.405	NA	NA	NA	NA
**Adjust for 2hGlu**	0.063	3.16E-01	0.506	0.921	1.78E-02	0.823	NA	NA	NA	NA
**Adjust for HbA1c**	0.038	**5.92E-04**	0.944	1.223	**3.08E-03**	0.183	0.046	0.576	1.78	7.31%
**Adjust for ALL**	NA	NA	NA	NA	NA	NA	0.376	0.253	1.29	59.78%

BMI, body mass index; TC, total cholesterol; TG, triglyceride; HDL, high density lipoprotein; LDL, low density lipoprotein; FG, fasting glucose; FI, fasting insulin; HOMA-β, homeostatic model assessment of beta cell function; HOMA-IR, homeostatic model assessment of insulin resistance; 2hGlu, 2-h postprandial glucose; HbA1c, hemoglobin; NA, not applicable; The IVW causal effect size was the beta coefficient estimated by IVW models for corresponding outcome; Direct causal effect: this value is obtained by subtracting the indirect effect from 0.629 as show in **Table 5**; IVW p values < 0.05/11 indicate statistical significance and are marked in bold font, and mediation analysis is significant only if both MR steps reach statistical significance; Proportion of mediation = Indirect causal effect by coefficient product/0.629.

**Table 5:** Cross-trait meta-analysis results between type 2 diabetes and television watching (*P*_meta_ < 2.5×10^-8^ and single-trait *P* < 1×10^-5^).

Index.SNP	CHR	Genome position	EA	NEA	EAF	T2D		TV watching		*P*_meta_	Genes	variant annotation
						BETA	P	BETA	P			
**rs4420638**	19	19q13.32	A	G	0.84	0.110	1.50E-09	0.014	3.60E-07	2.42E-14	[APOC1,APOE,PVRL2,TOMM40]	downstream
**rs4565329**	6	6q22.32	T	C	0.48	0.073	4.40E-09	0.010	1.50E-06	7.64E-14	[CENPW]	intron
**rs74333814**	11	11q13.4	T	C	0.86	-0.095	5.80E-09	-0.014	3.50E-06	3.84E-13	[ARAP1,ATG16L2,FCHSD2,MIR4692,STARD10]	intron
**rs243024**	2	2p16.1	A	G	0.55	0.066	3.90E-08	0.011	1.00E-06	4.39E-12	[AC007381.3]*	upstream
**rs2258238**	12	12q14.3	A	T	0.88	-0.110	1.60E-07	-0.016	7.40E-06	1.23E-11	[HMGA2,RPSAP52]	intron
**rs10400419**	12	12q14.3	T	C	0.57	-0.067	1.70E-07	-0.010	9.60E-06	1.34E-10	[HMGA2]*	intergenic
**rs550057**	9	9q34.2	T	C	0.76	0.065	3.40E-06	0.012	3.00E-06	1.81E-09	[ABO]	intron
**rs78028320**	11	11q13.1	A	G	0.82	0.069	5.80E-06	0.013	3.90E-06	2.68E-09	[CFL1]*	intergenic

EA, effect allele; NEA, noneffect allele; P_meta_ is the cross-trait meta-analysis P value. CHR, chromosome; T2D, type 2 diabetes; genes in * are the nearest genes to this locus.

Finally, we calculated the statistical power of this study using the mRnd website (64) (https://shiny.cnsgenomics.com/mRnd/). With the current sample size of T2D and the phenotypic variance of TV watching explained by IVs (4.1%, **Table S3**), at an alpha level of 0.05, we had 99% power to determine that each standard deviation increase in TV watching time increased the overall risk of T2D by 86% (i.e., an OR_IVW_ of 1.86, **Table 2**).

## Discussion

In the present study, we conducted a comprehensive genetic analysis to explore causal relationships and genetic overlap between T2D and TV watching and breakfast skipping by using summary statistics from GWAS. In the first instance, we showed that there was a strong positive genetic correlation between T2D and both exposures. Second, shared genetic structure at the locus level was identified between T2D and TV watching in cross-trait association analysis. Third, in the TWAS study between T2D and TV watching, we identified TWAS-significant genes, especially in tissues from the endocrine system, cardiovascular system, digestive system and nervous system. Finally, and most importantly, bidirectional MR showed that TV watching was positively associated with the risk of T2D. Mediation analysis identified four different traits as potential mediating factors between TV watching and T2D. Our results in the present study highlighted that TV watching plays an important role in the risk of T2D. The genetic overlaps elucidate potential shared biological pathways, thus providing new ideas and opportunities for T2D treatment and drug design.

The results of genetic correlation analysis are highly consistent with observational studies showing that breakfast skipping (8) and TV watching are significantly associated with an increased risk of T2D (4). These findings do not necessarily imply that TV watching per se causes T2D; rather, we believe that prolonged TV watching and breakfast skipping significantly affect the risk of developing diabetes in the future. There are two possible explanations for the observed positive association between TV watching and the risk of T2D. First, prolonged TV watching may result in lower energy expenditure and higher caloric intake, which are directly associated with obesity and weight gain (65, 66). Second, individuals who spend more time watching TV tend to eat more processed meats, snacks, and sweets and fewer vegetables and fruits, and such a diet may inversely affect diabetes risk (67). The average time spent watching TV is significantly associated with elevated levels of leptin and LDL cholesterol and lower levels of HDL cholesterol and apolipoprotein, which are important plasma biomarkers of T2D (68). Similarly, skipping breakfast may also trigger hyperglycemia and high glycated hemoglobin after lunch and dinner, further leading to impaired insulin response and thus increasing the risk of T2D (69). For these possible mechanistic pathways, we made presumptions and validated them in the subsequent shared genetic structure analysis and MR-mediated analysis.

CPASSOC and TWAS showed that the shared genes between TV watching and T2D were mostly enriched in the endocrine system and cardiovascular system, suggesting an underlying correlation between the biological pathway and these tissues. Study shows that the *CFL1* gene, which controls cell proliferation and cell death, is overexpressed in the subcutaneous adipose tissue of subjects who have gained weight, suggesting that the *CFL1* gene affects the risk of T2D through a mediating pathway of BMI (70). Reports have demonstrated that elevated *APOC1* gene expression is significantly associated with the risk of T2D and TG levels; also, apoC1 glycosylation has been observed in patients with T2D, which impairs the ability of *APOC1* to inhibit plasma cholesteryl ester transporter protein activity, suggesting that elevated apoC1 expression may increase the risk of T2D through lipoprotein metabolic pathways (71, 72). *APOC1* has also been reported to activate lecithin-cholesterol acyltransferase (LCAT), which in turn promotes HDL cholesterol esterification and increases HDL levels (73). Furthermore, increased *HMGA2* expression can be expected to lead to increased expression of p14^Arf^, an inducer of cellular senescence, and the accumulation of senescent cells triggers inflammation associated with insulin resistance, driving the development of T2D, predicting that TV watching induces a signaling pathway linked to cellular senescence to increase the risk of T2D (43). Of additional interest to us is the fact that individuals who watch television for long periods of time consume more food and energy, increasing the burden on the digestive system (74). Additionally, patients with T2D often experience gastrointestinal disturbances, suggesting that gastrointestinal disturbances play a collider role in the association between TV watching and T2D (75). The exact mechanism of the digestive system in this association needs to be further elaborated. Moreover, previous research shows that *APAR1* affects the function of pancreatic β-cells and that the proinsulin-raising allele of *ARAP1* is related to a decreasing risk of T2D (76). The opposite conclusion was also reported: T2D pathogenic activity is mediated by *STARD10* expression instead of *ARAP1* (77), but both genes are located in a specific region, 11q13.4, which was identified in our cross-trait analysis, implying that pancreatic β-cell and proinsulin processing may be located in the biological pathway between TV watching and T2D. Our study suggests that multisystem, multitissue, polygenic effects may have a synergistic effect on the risk of T2D, but this needs more experimental evidence for further clarification.

Overall, using the MR study design, we found strong causal relationship between TV watching time and an increased risk of T2D. The observed causal effect was greatly attenuated when the mediating role of BMI, glycemia, and lipids was taken into account, suggesting that BMI, glycemia, and lipids play a key role in the association. Our finding is consistent with most previous observational studies and meta-analyses showing that prolonged TV watching is associated with an increased risk of T2D. A recent systematic review and dose–response meta-analysis based on 11 prospective studies published from 2001-2016 showed a linear association between TV watching and T2D (78), which was again validated in a recent meta-analysis (79). Our results are also supported by previous epidemiological studies that used Cox proportional hazards regression, controlling for multiple time-independent (i.e., constant across all cycles) and time-related (i.e., varying from cycle to cycle) covariates, to clarify that watching more than 4 hours of television and video per day at age 16 increases the risk of developing T2D (80). Moreover, this association was also verified in a multivariate logistic regression study based on an East Asian population that took into account gender differences (6). In addition, cross-sectional and longitudinal studies assessing the association between TV watching time and cardiometabolic biomarkers among multiple ethnic groups corroborated the plausibility of our choice of mediating variables and provided some potential mechanistic pathways that act through these mediators (62, 68, 81). However, a recent MR analysis of sedentary behavior with T2D and glycemic traits contradicts our results, finding no causal relationship between sedentary behavior and T2D. Two reasons may explain this discrepancy, one of which is that sedentary behavior is assessed by accelerometers, which is not conducive to measuring posture and sedentariness and estimating energy expenditure (82). In addition, the presence of the Hawthorne effect makes it possible for subjects to change their habituation (83). Second, although they also used data from UKB, the sample size was so small (*N* = 91084) that they could not select enough IVs to improve the statistical power (number of IVs = 6 in their study) (84). We also acknowledge the discrepancy between the results of breakfast skipping and T2D, and the findings of traditional epidemiological investigations may be partly due to fewer IVs for breakfast skipping.

In contrast to traditional observational studies and randomized controlled trials, the highlight of this study is the MR approach, which allows estimation of the causal effect of unhealthy lifestyles on T2D with a large sample size and high precision, controlling for potential reverse causality and confounders to the maximum extent possible. In addition, this study used various methods for sensitivity analysis, especially excluding SNPs related to potential confounders, to enhance the strength of instrumental variables and improve the robustness of estimation. Two-step MR mediation analysis was used in our study. When the results are binary variables (e.g., T2D), the estimation accuracy obtained by this method is higher than that obtained by multivariate Mendelian randomization (MVMR) (85). However, several potential shortcomings need to be acknowledged. First, in TWAS and GWAS-eQTL analysis, small eQTL samples are not sufficient to detect relatively weak signals, reducing the efficacy of the method. Second, our study is limited to individuals of European ancestry and cannot be generalized to other ethnicities. Third, no sex-specific MR analysis was conducted for the association between TV watching and T2D in our study. In addition, the analysis of breakfast skipping was limited to a few IVs and could not produce results with high power and reliability. Finally, further exploration of unhealthy lifestyle and T2D association mechanisms in the future, such as larger replication studies, sex-specific studies based on individual data, and more studies of mediating factors (hypertension, physical activity, education attainment, diet, leptin level, etc.), would greatly benefit our findings.

Our comprehensive genetic analysis identified shared genetic similarities between TV watching and T2D, suggesting a strong intrinsic genetic link between this trait pair. We further used MR to find convincing evidence supporting a putative causal role between TV watching and T2D, but mediation analyses suggest that this effect is largely mediated by BMI, HbA1c, FG, and HDL. As obesity, hyperglycemia, and hyperlipidemia are recognized as established risk factors for T2D, our findings underscore the importance of actionable prevention strategies for T2D. However, to date, the complex interactions between TV watching and T2D do not appear to be fully understood, and further studies are needed to deepen our understanding of the biological pathways by which TV watching influences T2D.

## Data Availability Statement

The original contributions presented in the study are included in the article/**Supplementary Material**. Further inquiries can be directed to the corresponding authors. Custom ***R*** scripts used to generate results in this study can be made available upon request

## Ethics Statement

The study was conducted using publicly available summary-level genetic data, and no ethical approval was requested.

## Author Contributions

JJ and TH conceived and designed the study. DC and HW performed the data preparation and statistical analysis. DC and HW wrote the manuscript. DC and HW contributed equally to this article. All authors helped interpret the data, reviewed and edited the final paper and approved the submission.

## Conflict of Interest

The authors declare that the research was conducted in the absence of any commercial or financial relationships that could be construed as a potential conflict of interest.

## Publisher’s Note

All claims expressed in this article are solely those of the authors and do not necessarily represent those of their affiliated organizations, or those of the publisher, the editors and the reviewers. Any product that may be evaluated in this article, or claim that may be made by its manufacturer, is not guaranteed or endorsed by the publisher.

## Abbreviations

T2D, Type 2 diabetes; BMI, body mass index; SNV, Single nucleotide variant; SNP, Single nucleotide polymorphism; GWAS, genome-wide association study; HDL, High density lipoprotein; HDL-method, High-definition likelihood; LDSC, Linkage disequilibrium score regression; CPASSOC, Cross phenotype association study; eQTL, expression quantitative trait loci; MR, Mendelian randomization; TWAS, Transcriptome wide association study; GTEx, Genotype-tissue expression portal.

## References

[1] Meigs JB Cupples LA Wilson PW. Parental Transmission of Type 2 Diabetes: The Framingham Offspring Study. Diabetes (2000) 49:2201–7. doi: 10.2337/diabetes.49.12.2201 11118026

[2] Ali O. Genetics of Type 2 Diabetes. World J Diabetes (2013) 4:114–23. doi: 10.4239/wjd.v4.i4.114 PMC3746083 23961321

[3] Hu FB Li TY Colditz GA Willett WC Manson JE. Television Watching and Other Sedentary Behaviors in Relation to Risk of Obesity and Type 2 Diabetes Mellitus in Women. JAMA (2003) 289:1785–91. doi: 10.1001/jama.289.14.1785 12684356

[4] Grontved A Hu FB. Television Viewing and Risk of Type 2 Diabetes, Cardiovascular Disease, and All-Cause Mortality: A Meta-Analysis. JAMA (2011) 305:2448–55. doi: 10.1001/jama.2011.812 PMC4324728 21673296

[5] Bennett DA Du H Bragg F Guo Y Wright N Yang L. Physical Activity, Sedentary Leisure-Time and Risk of Incident Type 2 Diabetes: A Prospective Study of 512 000 Chinese Adults. BMJ Open Diabetes Res Care (2019) 7:e000835. doi: 10.1136/bmjdrc-2019-000835 PMC6936425 31908799

[6] Ikehara S Iso H Maruyama K Ukawa S Tamakoshi A. Television Viewing Time, Walking Time, and Risk of Type 2 Diabetes in Japanese Men and Women: The Japan Collaborative Cohort Study. Prev Med (2019) 118:220–5. doi: 10.1016/j.ypmed.2018.11.006 30408447

[7] Mekary RA Giovannucci E Willett WC Van Dam RM Hu FB. Eating Patterns and Type 2 Diabetes Risk in Men: Breakfast Omission, Eating Frequency, and Snacking. Am J Clin Nutr (2012) 95:1182–9. doi: 10.3945/ajcn.111.028209 PMC3325839 22456660

[8] Bi H Gan Y Yang C Chen Y Tong X Lu Z. Breakfast Skipping and the Risk of Type 2 Diabetes: A Meta-Analysis of Observational Studies. Public Health Nutr (2015) 18:3013–9. doi: 10.1017/S1368980015000257 PMC10271832 25686619

[9] Ballon A Neuenschwander M Schlesinger S. Breakfast Skipping Is Associated With Increased Risk of Type 2 Diabetes Among Adults: A Systematic Review and Meta-Analysis of Prospective Cohort Studies. J Nutr (2019) 149:106–13. doi: 10.1093/jn/nxy194 30418612

[10] Krishnan S Rosenberg L Palmer JR. Physical Activity and Television Watching in Relation to Risk of Type 2 Diabetes: The Black Women’s Health Study. Am J Epidemiol (2009) 169:428–34. doi: 10.1093/aje/kwn344 PMC2726641 19056835

[11] Al-Goblan AS Al-Alfi MA Khan MZ. Mechanism Linking Diabetes Mellitus and Obesity. Diabetes Metab Syndr Obes (2014) 7:587–91. doi: 10.2147/DMSO.S67400 PMC4259868 25506234

[12] Deshmukh-Taskar P Nicklas TA Radcliffe JD O’neil CE Liu Y. The Relationship of Breakfast Skipping and Type of Breakfast Consumed With Overweight/Obesity, Abdominal Obesity, Other Cardiometabolic Risk Factors and the Metabolic Syndrome in Young Adults. The National Health and Nutrition Examination Survey (NHANES): 1999-2006. Public Health Nutr (2013) 16:2073–82. doi: 10.1017/S1368980012004296 PMC10271246 23031568

[13] Haase CL Tybjærg-Hansen A Nordestgaard BG Frikke-Schmidt R. HDL Cholesterol and Risk of Type 2 Diabetes: A Mendelian Randomization Study. Diabetes (2015) 64:3328–33. doi: 10.2337/db14-1603 25972569

[14] Verduijn M Siegerink B Jager KJ Zoccali C Dekker FW. Mendelian Randomization: Use of Genetics to Enable Causal Inference in Observational Studies. Nephrol Dialysis Transplant (2010) 25:1394–8. doi: 10.1093/ndt/gfq098 20190244

[15] Mahajan A Wessel J Willems SM Zhao W Robertson NR Chu AY. Refining the Accuracy of Validated Target Identification Through Coding Variant Fine-Mapping in Type 2 Diabetes. Nat Genet (2018) 50:559–71. doi: 10.1038/s41588-018-0084-1 PMC5898373 29632382

[16] Dashti HS Merino J Lane JM Song Y Smith CE Tanaka T. Genome-Wide Association Study of Breakfast Skipping Links Clock Regulation With Food Timing. Am J Clin Nutr (2019) 110:473–84. doi: 10.1093/ajcn/nqz076 PMC6669061 31190057

[17] Van De Vegte YJ Said MA Rienstra M van der Harst P Verweij N. Genome-Wide Association Studies and Mendelian Randomization Analyses for Leisure Sedentary Behaviours. Nat Commun (2020) 11:1770. doi: 10.1038/s41467-020-15553-w 32317632 PMC7174427

[18] Yang F Chen S Qu Z Wang K Xie X Cui H. Genetic Liability to Sedentary Behavior in Relation to Stroke, Its Subtypes and Neurodegenerative Diseases: A Mendelian Randomization Study. Front Aging Neurosci (2021) 13:769. doi: 10.3389/fnagi.2021.757388 PMC8641575 34867285

[19] Park S Lee S Kim Y Lee Y Kang MW Kim K. Causal Effects of Physical Activity or Sedentary Behaviors on Kidney Function: An Integrated Population-Scale Observational Analysis and Mendelian Randomization Study. Nephrol Dialysis Transplant (2021). doi: 10.1093/ndt/gfab153 33826736

[20] Gao Y Mi J Liu Z Song Q. Leisure Sedentary Behavior and Risk of Lung Cancer: A Two-Sample Mendelian Randomization Study and Mediation Analysis. Front Genet (2021) 12. doi: 10.3389/fgene.2021.763626 PMC8582637 34777480

[21] Relton CL Davey Smith G. Mendelian Randomization: Applications and Limitations in Epigenetic Studies. Epigenomics (2015) 7:1239–43. doi: 10.2217/epi.15.88 PMC5330409 26639554

[22] Solovieff N Cotsapas C Lee PH Purcell SM Smoller JW. Pleiotropy in Complex Traits: Challenges and Strategies. Nat Rev Genet (2013) 14:483–95. doi: 10.1038/nrg3461 PMC4104202 23752797

[23] Scott RA Scott LJ Mägi R Marullo L Gaulton KJ Kaakinen M. An Expanded Genome-Wide Association Study of Type 2 Diabetes in Europeans. Diabetes (2017) 66:2888–902. doi: 10.2337/db16-1253 PMC5652602 28566273

[24] Ning Z Pawitan Y Shen X. High-Definition Likelihood Inference of Genetic Correlations Across Human Complex Traits. Nat Genet (2020) 52:859–64. doi: 10.1038/s41588-020-0653-y 32601477

[25] Bulik-Sullivan BK Loh PR Finucane HK Ripke S Yang J Patterson N. LD Score Regression Distinguishes Confounding From Polygenicity in Genome-Wide Association Studies. Nat Genet (2015) 47:291–5. doi: 10.1038/ng.3211 PMC4495769 25642630

[26] Zhu Z Hasegawa K Camargo CA Jr. Liang L. Investigating Asthma Heterogeneity Through Shared and Distinct Genetics: Insights From Genome-Wide Cross-Trait Analysis. J Allergy Clin Immunol (2021) 147:796–807. doi: 10.1016/j.jaci.2020.07.004 32693092 PMC7368660

[27] Zhu X Feng T Tayo BO Liang J Young JH Franceschini N. Meta-Analysis of Correlated Traits *via* Summary Statistics From GWASs With an Application in Hypertension. Am J Hum Genet (2015) 96:21–36. doi: 10.1016/j.ajhg.2014.11.011 25500260 PMC4289691

[28] Giambartolomei C Vukcevic D Schadt EE Franke L Hingorani AD Wallace C. Bayesian Test for Colocalisation Between Pairs of Genetic Association Studies Using Summary Statistics. PloS Genet (2014) 10:e1004383. doi: 10.1371/journal.pgen.1004383 24830394 PMC4022491

[29] Carithers LJ Ardlie K Barcus M Branton PA Britton A Buia SA. A Novel Approach to High-Quality Postmortem Tissue Procurement: The GTEx Project. Biopreserv Biobank (2015) 13:311–9. doi: 10.1089/bio.2015.0032 PMC4675181 26484571

[30] Gusev A Ko A Shi H Bhatia G Chung W Penninx BW. Integrative Approaches for Large-Scale Transcriptome-Wide Association Studies. Nat Genet (2016) 48:245–52. doi: 10.1038/ng.3506 PMC4767558 26854917

[31] Mahajan A Taliun D Thurner M Robertson NR Torres JM Rayner NW. Fine-Mapping Type 2 Diabetes Loci to Single-Variant Resolution Using High-Density Imputation and Islet-Specific Epigenome Maps. Nat Genet (2018) 50:1505–13. doi: 10.1038/s41588-018-0241-6 PMC6287706 30297969

[32] Bowden J Davey Smith G Haycock PC Burgess S. Consistent Estimation in Mendelian Randomization With Some Invalid Instruments Using a Weighted Median Estimator. Genet Epidemiol (2016) 40:304–14. doi: 10.1002/gepi.21965 PMC4849733 27061298

[33] Bowden J Davey Smith G Burgess S. Mendelian Randomization With Invalid Instruments: Effect Estimation and Bias Detection Through Egger Regression. Int J Epidemiol (2015) 44:512–25. doi: 10.1093/ije/dyv080 PMC4469799 26050253

[34] Hemani G Tilling K Davey Smith G. Orienting the Causal Relationship Between Imprecisely Measured Traits Using GWAS Summary Data. PloS Genet (2017) 13:e1007081. doi: 10.1371/journal.pgen.1007081 29149188 PMC5711033

[35] Zhao Q Wang J Hemani G Bowden J Small DS. Statistical Inference in Two-Sample Summary-Data Mendelian Randomization Using Robust Adjusted Profile Score. Ann Stat (2020) 48:1742–69. doi: 10.1214/19-AOS1866

[36] Noyce AJ Kia DA Hemani G Nicolas A Price TR De Pablo-Fernandez E. Estimating the Causal Influence of Body Mass Index on Risk of Parkinson Disease: A Mendelian Randomisation Study. PloS Med (2017) 14:e1002314. doi: 10.1371/journal.pmed.1002314 28609445 PMC5469450

[37] Verbanck M Chen CY Neale B Do R. Detection of Widespread Horizontal Pleiotropy in Causal Relationships Inferred From Mendelian Randomization Between Complex Traits and Diseases. Nat Genet (2018) 50:693–8. doi: 10.1038/s41588-018-0099-7 PMC6083837 29686387

[38] Carter AR Sanderson E Hammerton G Richmond RC Davey Smith G Heron J. Mendelian Randomisation for Mediation Analysis: Current Methods and Challenges for Implementation. Eur J Epidemiol (2021) 36:465–78. doi: 10.1007/s10654-021-00757-1 PMC8159796 33961203

[39] Manning AK Hivert MF Scott RA Grimsby JL Bouatia-Naji N Chen H. A Genome-Wide Approach Accounting for Body Mass Index Identifies Genetic Variants Influencing Fasting Glycemic Traits and Insulin Resistance. Nat Genet (2012) 44:659–69. doi: 10.1038/ng.2274 PMC3613127 22581228

[40] Willer CJ Schmidt EM Sengupta S Peloso GM Gustafsson S Kanoni S. Discovery and Refinement of Loci Associated With Lipid Levels. Nat Genet (2013) 45:1274–83. doi: 10.1038/ng.2797 PMC3838666 24097068

[41] Turcot V Lu Y Highland HM Schurmann C Justice AE Fine RS. Protein-Altering Variants Associated With Body Mass Index Implicate Pathways That Control Energy Intake and Expenditure in Obesity. Nat Genet (2018) 50:26–41. doi: 10.1038/s41588-019-0447-2 29273807 PMC5945951

[42] Chen J Spracklen CN Marenne G Varshney A Corbin LJ Luan J. The Trans-Ancestral Genomic Architecture of Glycemic Traits. Nat Genet (2021) 53:840–60. doi: 10.1038/s41588-021-00852-9 PMC7610958 34059833

[43] Markowski DN Thies HW Gottlieb A Wenk H Wischnewsky M Bullerdiek J. HMGA2 Expression in White Adipose Tissue Linking Cellular Senescence With Diabetes. Genes Nutr (2013) 8:449–56. doi: 10.1007/s12263-013-0354-6 PMC3755135 23881689

[44] Wasik AA Koskelainen S Hyvönen ME Musante L Lehtonen E Koskenniemi K. Ezrin Is Down-Regulated in Diabetic Kidney Glomeruli and Regulates Actin Reorganization and Glucose Uptake *via* GLUT1 in Cultured Podocytes. Am J Pathol (2014) 184:1727–39. doi: 10.1016/j.ajpath.2014.03.002 24726496

[45] Pagtalunan ME Miller PL Jumping-Eagle S Nelson RG Myers BD Rennke HG. Podocyte Loss and Progressive Glomerular Injury in Type II Diabetes. J Clin Invest (1997) 99:342–8. doi: 10.1172/JCI119163 PMC507802 9006003

[46] Garg P Verma R Cook L Soofi A Venkatareddy M George B. Actin-Depolymerizing Factor Cofilin-1 Is Necessary in Maintaining Mature Podocyte Architecture. J Biol Chem (2010) 285:22676–88. doi: 10.1074/jbc.M110.122929 PMC2903407 20472933

[47] Xiao H Xu Y. Overexpression of Apolipoprotein C1 (APOC1) in Clear Cell Renal Cell Carcinoma and Its Prognostic Significance. Med Sci Monit (2021) 27:e929347. doi: 10.12659/MSM.929347 33591959 PMC7896428

[48] Kasthuri RS Mcmillan KR Flood-Urdangarin C Harvey SB Wilson-Grady JT Nelsestuen GL. Correlation of a T45S Variant of Apolipoprotein C1 With Elevated BMI in Persons of American Indian and Mexican Ancestries. Int J Obes (Lond) (2007) 31:1334–6. doi: 10.1038/sj.ijo.0803569 17310220

[49] Zhao W Rasheed A Tikkanen E Lee JJ Butterworth AS Howson JMM. Identification of New Susceptibility Loci for Type 2 Diabetes and Shared Etiological Pathways With Coronary Heart Disease. Nat Genet (2017) 49:1450–7. doi: 10.1038/ng.3943 PMC5844224 28869590

[50] Carrat G Meur G Rutter G. Roles of the Type 2 Diabetes Associated Gene Products Arap1 and StarD10 in the Control of Insulin Secretion. Diabetic Med (2013) 30:31–1. doi: 10.1016/j.molmet.2020.101015

[51] Winkler TW Justice AE Graff M Barata L Feitosa MF Chu S. The Influence of Age and Sex on Genetic Associations With Adult Body Size and Shape: A Large-Scale Genome-Wide Interaction Study. PloS Genet (2015) 11:e1005378. doi: 10.1371/journal.pgen.1006166 26426971 PMC4591371

[52] Locke AE Kahali B Berndt SI Justice AE Pers TH Day FR. Genetic Studies of Body Mass Index Yield New Insights for Obesity Biology. Nature (2015) 518:197–206. doi: 10.1038/nature14177 25673413 PMC4382211

[53] Greenwell P. Blood Group Antigens: Molecules Seeking a Function? Glycoconj J (1997) 14:159–73. doi: 10.1023/A:1018581503164 9111133

[54] Kim J Kraft P Hagan KA Harrington LB Lindstroem S Kabrhel C. Interaction of a Genetic Risk Score With Physical Activity, Physical Inactivity, and Body Mass Index in Relation to Venous Thromboembolism Risk. Genet Epidemiol (2018) 42:354–65. doi: 10.1002/gepi.22118 PMC5980715 29520861

[55] Qi L Cornelis MC Kraft P Jensen M Van Dam RM Sun Q. Genetic Variants in ABO Blood Group Region, Plasma Soluble E-Selectin Levels and Risk of Type 2 Diabetes. Hum Mol Genet (2010) 19:1856–62. doi: 10.1093/hmg/ddq057 PMC2850622 20147318

[56] Caberlotto L Nguyen TP Lauria M Priami C Rimondini R Maioli S. Cross-Disease Analysis of Alzheimer’s Disease and Type-2 Diabetes Highlights the Role of Autophagy in the Pathophysiology of Two Highly Comorbid Diseases. Sci Rep (2019) 9:3965. doi: 10.1038/s41598-019-39828-5 30850634 PMC6408545

[57] Burgess S Thompson SG. Avoiding Bias From Weak Instruments in Mendelian Randomization Studies. Int J Epidemiol (2011) 40:755–64. doi: 10.1093/ije/dyr036 21414999

[58] Shim H Chasman DI Smith JD Mora S Ridker PM Nickerson DA. A Multivariate Genome-Wide Association Analysis of 10 LDL Subfractions, and Their Response to Statin Treatment, in 1868 Caucasians. PloS One (2015) 10:e0120758. doi: 10.1371/journal.pone.0120758 25898129 PMC4405269

[59] Staiger D Stock JH. Instrumental Variables Regression With Weak Instruments. Econometrica (1997) 65:557–86. doi: 10.2307/2171753

[60] Al-Ghamdi SH. The Association Between Watching Television and Obesity in Children of School-Age in Saudi Arabia. J Family Community Med (2013) 20:83–9. doi: 10.4103/2230-8229.114767 PMC3748652 23983559

[61] Martinez-Gomez D Rey-López JP Chillón P Gómez-Martínez S Vicente-Rodríguez G Martín-Matillas M. Excessive TV Viewing and Cardiovascular Disease Risk Factors in Adolescents. The AVENA Cross-Sectional Study. BMC Public Health (2010) 10:274–4. doi: 10.1186/1471-2458-10-274 PMC2892447 20500845

[62] Dunstan DW Salmon J Healy GN Shaw JE Jolley D Zimmet PZ. Association of Television Viewing With Fasting and 2-H Postchallenge Plasma Glucose Levels in Adults Without Diagnosed Diabetes. Diabetes Care (2007) 30:516–22. doi: 10.2337/dc06-1996 17327314

[63] Antwi J Lavin R Sullivan S Bellavia M. Perception of and Risk Factors for Type 2 Diabetes Among Students Attending an Upstate New York College: A Pilot Study. Diabetol Metab Syndr (2020) 12:25. doi: 10.1186/s13098-020-00535-1 32256715 PMC7106855

[64] Brion MJ Shakhbazov K Visscher PM. Calculating Statistical Power in Mendelian Randomization Studies. Int J Epidemiol (2013) 42:1497–501. doi: 10.1093/ije/dyt179 PMC3807619 24159078

[65] Hu FB Leitzmann MF Stampfer MJ Colditz GA Willett WC Rimm EB. Physical Activity and Television Watching in Relation to Risk for Type 2 Diabetes Mellitus in Men. Arch Intern Med (2001) 161:1542–8. doi: 10.1001/archinte.161.12.1542 11427103

[66] Vereecken CA Todd J Roberts C Mulvihill C Maes L. Television Viewing Behaviour and Associations With Food Habits in Different Countries. Public Health Nutr (2006) 9:244–50. doi: 10.1079/PHN2005847 16571179

[67] Hu FB. Sedentary Lifestyle and Risk of Obesity and Type 2 Diabetes. Lipids (2003) 38:103–8. doi: 10.1007/s11745-003-1038-4 12733740

[68] Nang EEK Salim A Wu Y Tai ES Lee J Van Dam RM. Television Screen Time, But Not Computer Use and Reading Time, is Associated With Cardio-Metabolic Biomarkers in a Multiethnic Asian Population: A Cross-Sectional Study. Int J Behav Nutr Phys Act (2013) 10:70. doi: 10.1186/1479-5868-10-70 23718927 PMC3680020

[69] Jakubowicz D Wainstein J Ahren B Landau Z Bar-Dayan Y Froy O. Fasting Until Noon Triggers Increased Postprandial Hyperglycemia and Impaired Insulin Response After Lunch and Dinner in Individuals With Type 2 Diabetes: A Randomized Clinical Trial. Diabetes Care (2015) 38:1820–6. doi: 10.2337/dc15-0761 26220945

[70] Sales V Patti M-E. The Ups and Downs of Insulin Resistance and Type 2 Diabetes: Lessons From Genomic Analyses in Humans. Curr Cardiovasc Risk Rep (2013) 7:46–59. doi: 10.1007/s12170-012-0283-8 23459395 PMC3583548

[71] Bouillet B Gautier T Blache D Pais De Barros J-P Duvillard L Petit J-M. Glycation of Apolipoprotein C1 Impairs Its CETP Inhibitory Property: Pathophysiological Relevance in Patients With Type 1 and Type 2 Diabetes. Diabetes Care (2014) 37:1148–56. doi: 10.2337/dc13-1467 24574346

[72] Bouillet B Gautier T Aho LS Duvillard L Petit JM Lagrost L. Plasma Apolipoprotein C1 Concentration is Associated With Plasma Triglyceride Concentration, But Not Visceral Fat, in Patients With Type 2 Diabetes. Diabetes Metab (2016) 42:263–6. doi: 10.1016/j.diabet.2016.01.003 26934823

[73] Soutar AK Garner CW Baker HN Sparrow JT Jackson RL Gotto AM. Effect of the Human Plasma Apolipoproteins and Phosphatidylcholine Acyl Donor on the Activity of Lecithin: Cholesterol Acyltransferase. Biochemistry (1975) 14:3057–64. doi: 10.1021/bi00685a003 167813

[74] Temple JL Giacomelli AM Kent KM Roemmich JN Epstein LH. Television Watching Increases Motivated Responding for Food and Energy Intake in Children. Am J Clin Nutr (2007) 85:355–61. doi: 10.1093/ajcn/85.2.355 17284729

[75] Du YT Rayner CK Jones KL Talley NJ Horowitz M. Gastrointestinal Symptoms in Diabetes: Prevalence, Assessment, Pathogenesis, and Management. Diabetes Care (2018) 41:627–37. doi: 10.2337/dc17-1536 29463666

[76] Kulzer JR Stitzel ML Morken MA Huyghe JR Fuchsberger C Kuusisto J. A Common Functional Regulatory Variant at a Type 2 Diabetes Locus Upregulates ARAP1 Expression in the Pancreatic Beta Cell. Am J Hum Genet (2014) 94:186–97. doi: 10.1016/j.ajhg.2013.12.011 PMC3928648 24439111

[77] Carrat GR Hu M Nguyen-Tu M-S Chabosseau P Gaulton KJ Van De Bunt M. Decreased STARD10 Expression Is Associated With Defective Insulin Secretion in Humans and Mice. Am J Hum Genet (2017) 100:238–56. doi: 10.1016/j.ajhg.2017.01.011 PMC5294761 28132686

[78] Patterson R Mcnamara E Tainio M De Sá TH Smith AD Sharp SJ. Sedentary Behaviour and Risk of All-Cause, Cardiovascular and Cancer Mortality, and Incident Type 2 Diabetes: A Systematic Review and Dose Response Meta-Analysis. Eur J Epidemiol (2018) 33:811–29. doi: 10.1007/s10654-018-0380-1 PMC6133005 29589226

[79] Guo C Zhou Q Zhang D Qin P Li Q Tian G. Association of Total Sedentary Behaviour and Television Viewing With Risk of Overweight/Obesity, Type 2 Diabetes and Hypertension: A Dose-Response Meta-Analysis. Diabetes Obes Metab (2020) 22:79–90. doi: 10.1111/dom.13867 31468597

[80] Scandiffio JA Janssen I. Do Adolescent Sedentary Behavior Levels Predict Type 2 Diabetes Risk in Adulthood? BMC Public Health (2021) 21:969. doi: 10.1186/s12889-021-10948-w 34022833 PMC8140492

[81] Li C Beech B Crume T D’agostino RB Jr. Dabelea D Kaar JL. Longitudinal Association Between Television Watching and Computer Use and Risk Markers in Diabetes in the SEARCH for Diabetes in Youth Study. Pediatr Diabetes (2015) 16:382–91. doi: 10.1111/pedi.12163 PMC4291304 25041407

[82] Lee IM Shiroma EJ. Using Accelerometers to Measure Physical Activity in Large-Scale Epidemiological Studies: Issues and Challenges. Br J Sports Med (2014) 48:197–201. doi: 10.1136/bjsports-2013-093154 24297837 PMC3947179

[83] Pedišić Ž Bauman A. Accelerometer-Based Measures in Physical Activity Surveillance: Current Practices and Issues. Br J Sports Med (2015) 49:219–23. doi: 10.1136/bjsports-2013-093407 25370153

[84] Meisinger C Linseisen J Leitzmann M Baurecht H Baumeister SE. Association of Physical Activity and Sedentary Behavior With Type 2 Diabetes and Glycemic Traits: A Two-Sample Mendelian Randomization Study. BMJ Open Diabetes Res Care (2020) 8. doi: 10.1136/bmjdrc-2020-001896 PMC7725078 33293297

[85] Vanderweele TJ Vansteelandt S. Odds Ratios for Mediation Analysis for a Dichotomous Outcome. Am J Epidemiol (2010) 172:1339–48. doi: 10.1093/aje/kwq332 PMC2998205 21036955

