# Supplementary material for: Shared Genetic Basis and Causal Relationship Between Television Watching, Breakfast Skipping and Type 2 Diabetes: Evidence From a Comprehensive Genetic Analysis
Source: Front Endocrinol (Lausanne). 2022 Mar 24;13:836023. doi: 10.3389/fendo.2022.836023 (PMC8988136; doi:10.3389/fendo.2022.836023)

**Shared genetic basis and causal relationship between television watching, breakfast skipping and type 2 diabetes: evidence from a comprehensive genetic analysis**

Dongze Chen^1^, Hanyu Wu^2^, Xinpei Wang^1^, Tao Huang^3,4#^ and Jinzhu Jia^1,5#^

**Contents**

[Supplementary Table 2](#_Toc89796446)

[Table S1. List of instrucment variables used from the T2D to TV watching 2](#_Toc89796447)

[Table S2. List of instrucment variables used from the T2D to breakfast skipping 4](#_Toc89796448)

[Table S3. List of instrucment variables used from the TV watching to T2D 5](#_Toc89796449)

[Table S4. List of instrucment variables used from the breakfast skipping to T2D 11](#_Toc89796450)

[Table S5. List of SNPs with genome-wide significance for potential confounding traits in TV watching IVs searched from GWAS catelog 11](#_Toc89796451)

[Supplementary Figure 13](#_Toc89796452)

[Figure S1: Leave-one-out plot for univariable analysis of TV watching on T2D 13](#_Toc89796453)

[Figure S2: Leave-one-out plot for univariable analysis of breakfast skipping on T2D 14](#_Toc89796454)

[Figure S3: Leave-one-out plot for univariable analysis of T2D on TV watching 15](#_Toc89796455)

[Figure S4: Leave-one-out plot for univariable analysis of T2D on breakfast skipping 16](#_Toc89796456)

[Figure S5: Funnel plot assessing the extent to which pleiotropy is balanced across the set of instruments used in the univariable MR analysis of TV watching on T2D 17](#_Toc89796457)

[Figure S6: Funnel plot assessing the extent to which pleiotropy is balanced across the set of instruments used in the univariable MR analysis of breakfast skipping on T2D 18](#_Toc89796458)

[Figure S7: Funnel plot assessing the extent to which pleiotropy is balanced across the set of instruments used in the univariable MR analysis of T2D on TV watching 19](#_Toc89796459)

[Figure S8: Funnel plot assessing the extent to which pleiotropy is balanced across the set of instruments used in the univariable MR analysis of T2D on breakfast skipping 19](#_Toc89796460)

# Supplementary Table

## Table S1. List of instrucment variables used from the T2D to TV watching

| SNP | CHR | BP | A1 | A2 | EAF | T2D | | | TV watching | | | r2 |
| --- | --- | --- | --- | --- | --- | --- | --- | --- | --- | --- | --- | --- |
|  |  |  |  |  |  | BETA | SE | P | BETA | SE | P |  |
| rs10193447 | 2 | 60552476 | T | C | 0.603286 | 0.071 | 0.012 | 1.3E-08 | 0.006055 | 0.0022 | 0.0059 | 0.00022 |
| rs1061810 | 11 | 43877934 | A | C | 0.694807 | 0.08 | 0.014 | 5.3E-09 | -0.00167 | 0.002378 | 0.48 | 0.000205 |
| rs10882098 | 10 | 94444793 | T | C | 0.606606 | -0.13 | 0.012 | 1.4E-26 | 0.003161 | 0.002203 | 0.15 | 0.000737 |
| rs10965223 | 9 | 22067004 | A | G | 0.623428 | 0.077 | 0.012 | 4E-10 | -0.00086 | 0.002204 | 0.7 | 0.000259 |
| rs10965250 | 9 | 22133284 | A | G | 0.823828 | -0.14 | 0.016 | 2.7E-17 | 0.000206 | 0.002867 | 0.94 | 0.000481 |
| rs11257659 | 10 | 12309269 | T | C | 0.786699 | 0.081 | 0.015 | 2.7E-08 | 0.000278 | 0.002725 | 0.92 | 0.000183 |
| rs11616380 | 13 | 80705315 | T | G | 0.724743 | -0.09 | 0.014 | 3.9E-11 | 0.001272 | 0.002401 | 0.6 | 0.00026 |
| rs11708067 | 3 | 1.23E+08 | A | G | 0.787949 | 0.11 | 0.015 | 8.8E-13 | -0.001 | 0.002507 | 0.69 | 0.000338 |
| rs11759026 | 6 | 1.27E+08 | A | G | 0.752148 | -0.091 | 0.015 | 5.8E-10 | 0.008576 | 0.002588 | 0.00092 | 0.000231 |
| rs1635852 | 7 | 28189411 | T | C | 0.4944 | 0.092 | 0.012 | 3E-14 | -0.00157 | 0.002159 | 0.47 | 0.000369 |
| rs2023681 | 22 | 30599562 | A | G | 0.885305 | -0.12 | 0.021 | 3.9E-09 | -0.00386 | 0.003732 | 0.3 | 0.000205 |
| rs2215383 | 7 | 15062983 | T | C | 0.553221 | -0.069 | 0.012 | 1.4E-08 | -0.00117 | 0.002171 | 0.59 | 0.000208 |
| rs2237895 | 11 | 2857194 | A | C | 0.607117 | -0.097 | 0.013 | 1.7E-13 | 0.001499 | 0.002187 | 0.49 | 0.00035 |
| rs231360 | 11 | 2692249 | T | C | 0.432167 | 0.079 | 0.013 | 9.5E-10 | 0.00407 | 0.002221 | 0.067 | 0.000232 |
| rs28650790 | 5 | 55861464 | T | C | 0.802185 | 0.1 | 0.016 | 7.4E-10 | 0.001281 | 0.002757 | 0.64 | 0.000245 |
| rs340874 | 1 | 2.14E+08 | T | C | 0.471073 | -0.068 | 0.012 | 3.4E-08 | 0.00244 | 0.002177 | 0.26 | 0.000202 |
| rs35352848 | 3 | 23455582 | T | C | 0.80562 | 0.083 | 0.015 | 1.5E-08 | -0.00543 | 0.002672 | 0.042 | 0.000192 |
| rs3802177 | 8 | 1.18E+08 | A | G | 0.706622 | -0.11 | 0.013 | 1.7E-17 | -0.00011 | 0.002337 | 0.96 | 0.00045 |
| rs3821943 | 4 | 6299940 | T | C | 0.518884 | 0.1 | 0.012 | 4.2E-16 | 0.003132 | 0.002178 | 0.15 | 0.000436 |
| rs4238013 | 12 | 4376089 | T | C | 0.7944 | -0.099 | 0.017 | 3.6E-09 | 0.005359 | 0.002715 | 0.048 | 0.000213 |
| rs429358 | 19 | 45411941 | T | C | 0.862135 | 0.12 | 0.019 | 1.4E-10 | 0.013781 | 0.002982 | 3.8E-06 | 0.00025 |
| rs4402960 | 3 | 1.86E+08 | T | G | 0.671377 | 0.14 | 0.013 | 2.7E-25 | 0.005571 | 0.002327 | 0.017 | 0.000728 |
| rs4774420 | 15 | 62117975 | T | C | 0.701652 | -0.075 | 0.013 | 2.7E-08 | 0.006749 | 0.002364 | 0.0043 | 0.000209 |
| rs4846569 | 1 | 2.2E+08 | T | C | 0.720837 | -0.077 | 0.013 | 8.8E-09 | 0.002912 | 0.002371 | 0.22 | 0.00022 |
| rs5219 | 11 | 17409572 | T | C | 0.636295 | 0.068 | 0.012 | 4.3E-08 | -0.00132 | 0.002255 | 0.56 | 0.000202 |
| rs62530366 | 8 | 1.46E+08 | A | G | 0.595714 | -0.076 | 0.013 | 1.9E-08 | 0.00625 | 0.002235 | 0.0052 | 0.000215 |
| rs6757251 | 2 | 43734847 | T | C | 0.901147 | -0.13 | 0.021 | 1.9E-10 | 0.002559 | 0.003457 | 0.46 | 0.000241 |
| rs7428936 | 3 | 64710850 | T | C | 0.454982 | 0.07 | 0.012 | 1E-08 | 0.001012 | 0.002198 | 0.65 | 0.000214 |
| rs7451008 | 6 | 20673880 | T | C | 0.724157 | -0.17 | 0.013 | 3.8E-37 | 0.000298 | 0.002456 | 0.9 | 0.001073 |
| rs757209 | 17 | 36102833 | A | G | 0.584078 | -0.083 | 0.014 | 1.1E-09 | -0.00176 | 0.002205 | 0.42 | 0.000221 |
| rs76550717 | 11 | 72428172 | A | G | 0.861715 | 0.096 | 0.016 | 3.8E-09 | -0.01333 | 0.002953 | 6.4E-06 | 0.000226 |
| rs7903146 | 10 | 1.15E+08 | T | C | 0.692671 | 0.29 | 0.013 | 9.3E-108 | 0.000301 | 0.002378 | 0.9 | 0.003116 |
| rs8056814 | 16 | 75252327 | A | G | 0.908376 | -0.15 | 0.023 | 3.7E-11 | -0.00068 | 0.003915 | 0.86 | 0.000267 |
| rs810517 | 10 | 80942620 | T | C | 0.539353 | -0.089 | 0.013 | 1.3E-12 | -0.00139 | 0.002167 | 0.52 | 0.000294 |
| rs9410573 | 9 | 84311800 | T | C | 0.561091 | 0.073 | 0.013 | 2E-08 | -0.00071 | 0.002189 | 0.75 | 0.000198 |

## Table S2. List of instrucment variables used from the T2D to breakfast skipping

| SNP | CHR | BP | A1 | A2 | EAF | T2D | | | breakfast skipping | | | r2 |
| --- | --- | --- | --- | --- | --- | --- | --- | --- | --- | --- | --- | --- |
|  |  |  |  |  |  | BETA | SE | P | BETA | SE | P |  |
| rs10193447 | 2 | 60552476 | T | C | 0.603286 | 0.071 | 0.012 | 1.3E-08 | -0.00353 | 0.002744 | 0.2 | 0.00022 |
| rs1061810 | 11 | 43877934 | A | C | 0.694807 | 0.08 | 0.014 | 5.3E-09 | -0.00321 | 0.002953 | 0.29 | 0.000205 |
| rs10882098 | 10 | 94444793 | T | C | 0.606606 | -0.13 | 0.012 | 1.4E-26 | 0.007643 | 0.002731 | 0.0052 | 0.000737 |
| rs10965223 | 9 | 22067004 | A | G | 0.623428 | 0.077 | 0.012 | 4E-10 | -0.00165 | 0.002739 | 0.55 | 0.000259 |
| rs10965250 | 9 | 22133284 | A | G | 0.823828 | -0.14 | 0.016 | 2.7E-17 | 0.00089 | 0.00356 | 0.77 | 0.000481 |
| rs11616380 | 13 | 80705315 | T | G | 0.724743 | -0.09 | 0.014 | 3.9E-11 | -0.00351 | 0.002979 | 0.24 | 0.00026 |
| rs11708067 | 3 | 1.23E+08 | A | G | 0.787949 | 0.11 | 0.015 | 8.8E-13 | -0.00175 | 0.003121 | 0.55 | 0.000338 |
| rs11759026 | 6 | 1.27E+08 | A | G | 0.752148 | -0.091 | 0.015 | 5.8E-10 | 0.005658 | 0.003208 | 0.091 | 0.000231 |
| rs1635852 | 7 | 28189411 | T | C | 0.4944 | 0.092 | 0.012 | 3E-14 | 0.002872 | 0.00268 | 0.3 | 0.000369 |
| rs2023681 | 22 | 30599562 | A | G | 0.885305 | -0.12 | 0.021 | 3.9E-09 | -0.01064 | 0.004635 | 0.022 | 0.000205 |
| rs2215383 | 7 | 15062983 | T | C | 0.553221 | -0.069 | 0.012 | 1.4E-08 | -0.0039 | 0.002696 | 0.15 | 0.000208 |
| rs2237895 | 11 | 2857194 | A | C | 0.607117 | -0.097 | 0.013 | 1.7E-13 | 0.005871 | 0.002714 | 0.028 | 0.00035 |
| rs231360 | 11 | 2692249 | T | C | 0.432167 | 0.079 | 0.013 | 9.5E-10 | 0.000949 | 0.002759 | 0.74 | 0.000232 |
| rs28650790 | 5 | 55861464 | T | C | 0.802185 | 0.1 | 0.016 | 7.4E-10 | -0.00166 | 0.00341 | 0.61 | 0.000245 |
| rs340874 | 1 | 2.14E+08 | T | C | 0.471073 | -0.068 | 0.012 | 3.4E-08 | 0.001388 | 0.002706 | 0.6 | 0.000202 |
| rs35352848 | 3 | 23455582 | T | C | 0.80562 | 0.083 | 0.015 | 1.5E-08 | 0.00505 | 0.003325 | 0.14 | 0.000192 |
| rs3802177 | 8 | 1.18E+08 | A | G | 0.706622 | -0.11 | 0.013 | 1.7E-17 | -0.00137 | 0.002903 | 0.63 | 0.00045 |
| rs3821943 | 4 | 6299940 | T | C | 0.518884 | 0.1 | 0.012 | 4.2E-16 | -0.00505 | 0.002705 | 0.06 | 0.000436 |
| rs4238013 | 12 | 4376089 | T | C | 0.7944 | -0.099 | 0.017 | 3.6E-09 | -0.00093 | 0.003374 | 0.78 | 0.000213 |
| rs429358 | 19 | 45411941 | T | C | 0.862135 | 0.12 | 0.019 | 1.4E-10 | 0.014693 | 0.003738 | 0.000098 | 0.00025 |
| rs4402960 | 3 | 1.86E+08 | T | G | 0.671377 | 0.14 | 0.013 | 2.7E-25 | -0.0013 | 0.002895 | 0.66 | 0.000728 |
| rs4774420 | 15 | 62117975 | T | C | 0.701652 | -0.075 | 0.013 | 2.7E-08 | -0.00405 | 0.002938 | 0.16 | 0.000209 |
| rs4846569 | 1 | 2.2E+08 | T | C | 0.720837 | -0.077 | 0.013 | 8.8E-09 | 0.005194 | 0.002954 | 0.077 | 0.00022 |
| rs5219 | 11 | 17409572 | T | C | 0.636295 | 0.068 | 0.012 | 4.3E-08 | 0.000558 | 0.002794 | 0.84 | 0.000202 |
| rs62530366 | 8 | 1.46E+08 | A | G | 0.595714 | -0.076 | 0.013 | 1.9E-08 | 0.000947 | 0.002778 | 0.75 | 0.000215 |
| rs6757251 | 2 | 43734847 | T | C | 0.901147 | -0.13 | 0.021 | 1.9E-10 | -0.00389 | 0.004299 | 0.35 | 0.000241 |
| rs7428936 | 3 | 64710850 | T | C | 0.454982 | 0.07 | 0.012 | 1E-08 | -0.00193 | 0.002727 | 0.49 | 0.000214 |
| rs7451008 | 6 | 20673880 | T | C | 0.724157 | -0.17 | 0.013 | 3.8E-37 | -0.00096 | 0.003052 | 0.79 | 0.001073 |
| rs757209 | 17 | 36102833 | A | G | 0.584078 | -0.083 | 0.014 | 1.1E-09 | 0.000463 | 0.002739 | 0.89 | 0.000221 |
| rs76550717 | 11 | 72428172 | A | G | 0.861715 | 0.096 | 0.016 | 3.8E-09 | -0.00316 | 0.003674 | 0.38 | 0.000226 |
| rs7903146 | 10 | 1.15E+08 | T | C | 0.692671 | 0.29 | 0.013 | 9.3E-108 | 0.000482 | 0.002957 | 0.87 | 0.003116 |
| rs8056814 | 16 | 75252327 | A | G | 0.908376 | -0.15 | 0.023 | 3.7E-11 | -0.00844 | 0.004817 | 0.085 | 0.000267 |
| rs810517 | 10 | 80942620 | T | C | 0.539353 | -0.089 | 0.013 | 1.3E-12 | -0.00073 | 0.00269 | 0.78 | 0.000294 |
| rs9410573 | 9 | 84311800 | T | C | 0.561091 | 0.073 | 0.013 | 2E-08 | -0.00288 | 0.002718 | 0.3 | 0.000198 |

## Table S3. List of instrucment variables used from the TV watching to T2D

| SNP | CHR | BP | A1 | A2 | EAF | TV watching | | | T2D | | | r2 | confounder |
| --- | --- | --- | --- | --- | --- | --- | --- | --- | --- | --- | --- | --- | --- |
|  |  |  |  |  |  | BETA | SE | P | BETA | SE | P |  |  |
| rs10041724 | 5 | 1.24E+08 | T | C | 0.69185 | 0.018093 | 0.002739 | 0.000274 | 0.0047 | 0.016 | 0.77 | 0.000274 | 0.000274 |
| rs10054327 | 5 | 1.48E+08 | G | A | 0.514652 | 0.017245 | 0.00219 | 0.000389 | -0.0005 | 0.012 | 0.97 | 0.000389 | 0.000389 |
| rs10189857 | 2 | 60713235 | A | G | 0.525641 | -0.02046 | 0.00218 | 0.000553 | -0.013 | 0.013 | 0.31 | 0.000553 | 0.000553 |
| rs1022785 | 14 | 29571653 | G | A | 0.850275 | 0.018007 | 0.003111 | 7.1E-09 | 0.029 | 0.017 | 0.093 | 0.00021 | 0.00021 |
| rs10234444 | 7 | 41828527 | G | A | 0.774725 | 0.015891 | 0.002865 | 2.9E-08 | -0.0028 | 0.017 | 0.87 | 0.000193 | 0.000193 |
| rs1031423 | 5 | 93276883 | T | C | 0.717949 | -0.01852 | 0.002626 | 1.8E-12 | -0.012 | 0.016 | 0.44 | 0.000312 | 0.000312 |
| rs10427502 | 21 | 40654840 | G | A | 0.764652 | 0.013696 | 0.002233 | 8.6E-10 | 0.0052 | 0.013 | 0.68 | 0.000236 | 0.000236 |
| rs10771746 | 12 | 30791864 | C | T | 0.727564 | -0.01428 | 0.002395 | 2.5E-09 | 0.018 | 0.013 | 0.17 | 0.000223 | 0.000223 |
| rs10772643 | 12 | 13415288 | C | T | 0.878663 | 0.0248 | 0.003497 | 1.3E-12 | 0.015 | 0.019 | 0.44 | 0.000316 | 0.000316 |
| rs10874772 | 1 | 93787770 | G | A | 0.539377 | 0.012677 | 0.002257 | 1.9E-08 | 0.0004 | 0.013 | 0.98 | 0.000198 | 0.000198 |
| rs10876864 | 12 | 56401085 | G | A | 0.548077 | -0.01334 | 0.002184 | 1E-09 | -0.033 | 0.012 | 0.0071 | 0.000234 | 0.000234 |
| rs10890123 | 1 | 74802123 | C | T | 0.75 | 0.014212 | 0.002571 | 3.3E-08 | 0.012 | 0.015 | 0.41 | 0.000192 | 0.000192 |
| rs10932837 | 2 | 2.21E+08 | C | T | 0.537546 | -0.01311 | 0.002162 | 1.3E-09 | 0.0053 | 0.012 | 0.66 | 0.000231 | 0.000231 |
| rs10994943 | 10 | 63591413 | T | G | 0.663462 | 0.012987 | 0.002191 | 3.1E-09 | 0.036 | 0.013 | 0.0042 | 0.000221 | 0.000221 |
| rs11020045 | 11 | 92487907 | A | C | 0.727106 | -0.013 | 0.002302 | 1.6E-08 | -0.017 | 0.013 | 0.19 | 0.0002 | 0.0002 |
| rs11130793 | 3 | 60884659 | C | T | 0.553571 | 0.012922 | 0.002211 | 5E-09 | -0.0056 | 0.013 | 0.66 | 0.000215 | 0.000215 |
| rs11201422 | 10 | 86924483 | T | C | 0.834707 | 0.012688 | 0.002299 | 3.4E-08 | 0.026 | 0.013 | 0.043 | 0.000191 | 0.000191 |
| rs11218575 | 11 | 1.22E+08 | C | T | 0.687271 | 0.015375 | 0.002187 | 2.1E-12 | 0.023 | 0.013 | 0.064 | 0.00031 | 0.00031 |
| rs11245482 | 10 | 1.27E+08 | T | C | 0.650641 | -0.01323 | 0.002221 | 2.6E-09 | 0.013 | 0.012 | 0.3 | 0.000223 | 0.000223 |
| rs1156541 | 18 | 39952989 | C | T | 0.722527 | 0.014451 | 0.002601 | 2.8E-08 | 0.0051 | 0.014 | 0.72 | 0.000194 | 0.000194 |
| rs11654952 | 17 | 4813799 | T | G | 0.789377 | -0.01718 | 0.003136 | 4.3E-08 | -0.007 | 0.019 | 0.7 | 0.000189 | 0.000189 |
| rs11657730 | 17 | 79372489 | C | T | 0.811355 | 0.012948 | 0.002265 | 1.1E-08 | -0.0086 | 0.017 | 0.61 | 0.000205 | 0.000205 |
| rs11689199 | 2 | 1.01E+08 | A | G | 0.678114 | 0.018464 | 0.002204 | 5.5E-17 | 0.03 | 0.013 | 0.019 | 0.00044 | 0.00044 |
| rs11714337 | 3 | 71582521 | G | A | 0.78663 | 0.014402 | 0.002189 | 4.7E-11 | 0.021 | 0.012 | 0.093 | 0.000272 | 0.000272 |
| rs11763734 | 7 | 1.26E+08 | A | C | 0.721154 | -0.01246 | 0.002162 | 8.2E-09 | 0.0032 | 0.012 | 0.79 | 0.000209 | 0.000209 |
| rs12105701 | 2 | 41737200 | C | T | 0.721612 | -0.01288 | 0.002208 | 5.4E-09 | -0.019 | 0.012 | 0.13 | 0.000214 | 0.000214 |
| rs12289262 | 11 | 12894758 | C | T | 0.798077 | -0.01373 | 0.002445 | 2E-08 | 0.0081 | 0.015 | 0.58 | 0.000198 | 0.000198 |
| rs1243182 | 10 | 21916728 | C | T | 0.68544 | -0.01859 | 0.002342 | 2E-15 | -0.0095 | 0.013 | 0.47 | 0.000396 | 0.000396 |
| rs12476388 | 2 | 1.16E+08 | C | T | 0.628205 | 0.013303 | 0.002374 | 2.1E-08 | -0.0048 | 0.013 | 0.72 | 0.000197 | 0.000197 |
| rs12491503 | 3 | 1.66E+08 | G | A | 0.539835 | -0.01426 | 0.002299 | 5.5E-10 | -0.012 | 0.013 | 0.37 | 0.000242 | 0.000242 |
| rs12541615 | 8 | 1.19E+08 | T | C | 0.894689 | -0.01752 | 0.0028 | 3.9E-10 | -0.026 | 0.016 | 0.095 | 0.000246 | 0.000246 |
| rs12554512 | 9 | 23352293 | T | C | 0.6337 | 0.020702 | 0.002193 | 3.8E-21 | -0.043 | 0.012 | 0.00055 | 0.000559 | 0.000559 |
| rs12725114 | 1 | 62432641 | G | A | 0.813645 | 0.015309 | 0.002711 | 1.6E-08 | 0.0072 | 0.015 | 0.63 | 0.0002 | 0.0002 |
| rs1278847 | 1 | 1.1E+08 | C | A | 0.708791 | 0.015923 | 0.002347 | 1.2E-11 | 0.021 | 0.013 | 0.11 | 0.000289 | 0.000289 |
| rs13029509 | 2 | 2.15E+08 | G | A | 0.702381 | -0.01833 | 0.002158 | 2E-17 | -0.011 | 0.012 | 0.36 | 0.000453 | 0.000453 |
| rs13107325 | 4 | 1.03E+08 | C | T | 0.965659 | -0.02917 | 0.004123 | 1.5E-12 | -0.017 | 0.029 | 0.57 | 0.000314 | 0.000314 |
| rs138256022 | 3 | 11603090 | C | T | 0.97848 | -0.03135 | 0.005653 | 2.9E-08 | -0.011 | 0.037 | 0.76 | 0.000193 | 0.000193 |
| rs141184308 | 9 | 86463339 | A | G | 0.989469 | 0.043261 | 0.007773 | 2.6E-08 | 0.024 | 0.068 | 0.72 | 0.000195 | 0.000195 |
| rs1421334 | 8 | 30865733 | A | C | 0.567766 | 0.017314 | 0.002183 | 2.2E-15 | 0.043 | 0.012 | 0.00051 | 0.000395 | 0.000395 |
| rs1563908 | 5 | 60437381 | A | G | 0.784799 | 0.016346 | 0.002226 | 2.1E-13 | 0.018 | 0.013 | 0.17 | 0.000338 | 0.000338 |
| rs17207890 | 11 | 95490754 | G | A | 0.752747 | 0.015694 | 0.002287 | 6.7E-12 | 0.029 | 0.013 | 0.03 | 0.000296 | 0.000296 |
| rs17512836 | 18 | 53194961 | T | C | 0.985348 | 0.042214 | 0.006846 | 7E-10 | -0.013 | 0.039 | 0.75 | 0.000239 | 0.000239 |
| rs17727474 | 10 | 1.27E+08 | C | T | 0.894689 | 0.017551 | 0.002961 | 3.1E-09 | -0.014 | 0.016 | 0.37 | 0.000221 | 0.000221 |
| rs17789218 | 6 | 1.01E+08 | T | C | 0.8837 | 0.018596 | 0.002514 | 1.4E-13 | -0.0061 | 0.015 | 0.69 | 0.000344 | 0.000344 |
| rs1889778 | 1 | 61066279 | C | T | 0.574176 | 0.012432 | 0.002167 | 9.6E-09 | 0.011 | 0.012 | 0.36 | 0.000207 | 0.000207 |
| rs2034768 | 3 | 93986371 | A | G | 0.507784 | 0.014736 | 0.002158 | 8.6E-12 | 0.043 | 0.012 | 0.00035 | 0.000293 | 0.000293 |
| rs2045147 | 10 | 56660437 | A | G | 0.599817 | 0.012669 | 0.002177 | 5.9E-09 | 0.023 | 0.012 | 0.056 | 0.000213 | 0.000213 |
| rs2073869 | 9 | 1.36E+08 | C | T | 0.794414 | 0.018611 | 0.002908 | 1.5E-10 | -0.021 | 0.016 | 0.19 | 0.000257 | 0.000257 |
| rs2164744 | 12 | 1.1E+08 | T | C | 0.589744 | -0.01286 | 0.002247 | 1.1E-08 | -0.0068 | 0.013 | 0.59 | 0.000206 | 0.000206 |
| rs2173650 | 12 | 1.18E+08 | G | T | 0.815934 | 0.017845 | 0.003044 | 4.6E-09 | 0.0034 | 0.017 | 0.84 | 0.000216 | 0.000216 |
| rs2184364 | 6 | 1.43E+08 | A | G | 0.797619 | 0.01564 | 0.002637 | 3E-09 | 0.0026 | 0.015 | 0.86 | 0.000221 | 0.000221 |
| rs2447098 | 17 | 2277720 | C | A | 0.607143 | -0.01495 | 0.002178 | 6.8E-12 | 0.025 | 0.012 | 0.04 | 0.000296 | 0.000296 |
| rs2460 | 15 | 53073084 | G | A | 0.649725 | -0.01529 | 0.002459 | 5E-10 | -0.023 | 0.014 | 0.11 | 0.000243 | 0.000243 |
| rs2584597 | 17 | 61941284 | T | C | 0.746795 | 0.0151 | 0.002396 | 2.9E-10 | 0.045 | 0.014 | 0.00097 | 0.000249 | 0.000249 |
| rs2616830 | 9 | 1721385 | G | A | 0.606685 | 0.016465 | 0.002166 | 2.9E-14 | 0.017 | 0.012 | 0.16 | 0.000363 | 0.000363 |
| rs262890 | 5 | 62930015 | A | G | 0.705586 | -0.01857 | 0.002356 | 3.2E-15 | 0.0028 | 0.013 | 0.83 | 0.00039 | 0.00039 |
| rs2717559 | 8 | 1.44E+08 | A | G | 0.663919 | 0.01243 | 0.002194 | 1.5E-08 | 0.013 | 0.013 | 0.33 | 0.000201 | 0.000201 |
| rs2787374 | 9 | 1.03E+08 | T | C | 0.560897 | 0.012493 | 0.002194 | 1.2E-08 | 0.014 | 0.012 | 0.24 | 0.000204 | 0.000204 |
| rs303753 | 18 | 21074922 | G | A | 0.816392 | -0.01446 | 0.002291 | 2.7E-10 | -0.035 | 0.013 | 0.0069 | 0.00025 | 0.00025 |
| rs34864022 | 9 | 22609110 | A | G | 0.973443 | -0.02638 | 0.004357 | 1.4E-09 | -0.0019 | 0.022 | 0.93 | 0.00023 | 0.00023 |
| rs35574015 | 16 | 15146061 | T | C | 0.688645 | -0.01316 | 0.002375 | 3E-08 | -0.0089 | 0.013 | 0.51 | 0.000193 | 0.000193 |
| rs374722 | 2 | 1.48E+08 | G | A | 0.755952 | 0.024493 | 0.003024 | 5.5E-16 | 0.03 | 0.017 | 0.071 | 0.000412 | 0.000412 |
| rs3754970 | 2 | 1.62E+08 | T | C | 0.559982 | -0.015 | 0.00217 | 4.8E-12 | 0.0046 | 0.012 | 0.71 | 0.0003 | 0.0003 |
| rs3796386 | 3 | 49899795 | G | A | 0.709249 | -0.02617 | 0.00218 | 3.2E-33 | -0.015 | 0.012 | 0.23 | 0.000905 | 0.000905 |
| rs4334769 | 4 | 1.3E+08 | G | T | 0.534341 | 0.012131 | 0.002166 | 2.1E-08 | -0.0056 | 0.012 | 0.65 | 0.000197 | 0.000197 |
| rs4382592 | 9 | 1.35E+08 | T | G | 0.542125 | 0.013672 | 0.002358 | 6.7E-09 | 0.022 | 0.013 | 0.11 | 0.000211 | 0.000211 |
| rs4435081 | 12 | 74331820 | C | T | 0.544872 | 0.01211 | 0.002167 | 2.3E-08 | -0.0093 | 0.012 | 0.44 | 0.000196 | 0.000196 |
| rs4523073 | 6 | 67550288 | A | G | 0.736264 | -0.01375 | 0.002211 | 5E-10 | -0.0069 | 0.012 | 0.58 | 0.000243 | 0.000243 |
| rs457335 | 22 | 29943125 | A | G | 0.732601 | 0.015072 | 0.002582 | 5.3E-09 | 0.04 | 0.015 | 0.006 | 0.000214 | 0.000214 |
| rs4577309 | 2 | 1.91E+08 | A | G | 0.614011 | 0.016004 | 0.002168 | 1.6E-13 | -0.0047 | 0.012 | 0.7 | 0.000342 | 0.000342 |
| rs4675246 | 2 | 2.03E+08 | G | T | 0.903388 | -0.01485 | 0.002676 | 2.9E-08 | 0.0072 | 0.015 | 0.64 | 0.000193 | 0.000193 |
| rs4775373 | 15 | 61476936 | T | C | 0.614927 | 0.013223 | 0.002257 | 4.7E-09 | 0.013 | 0.013 | 0.31 | 0.000216 | 0.000216 |
| rs4810315 | 20 | 40014674 | G | A | 0.526099 | -0.01281 | 0.002305 | 2.7E-08 | -0.012 | 0.013 | 0.35 | 0.000194 | 0.000194 |
| rs4845364 | 1 | 1.54E+08 | A | G | 0.730769 | -0.0153 | 0.002156 | 1.3E-12 | 0.011 | 0.012 | 0.34 | 0.000316 | 0.000316 |
| rs4950109 | 1 | 98680037 | T | C | 0.568681 | -0.01279 | 0.002225 | 9E-09 | 0.0099 | 0.013 | 0.45 | 0.000207 | 0.000207 |
| rs4973576 | 2 | 2.34E+08 | C | A | 0.783883 | -0.01452 | 0.002379 | 1E-09 | -0.0087 | 0.013 | 0.52 | 0.000234 | 0.000234 |
| rs55700114 | 20 | 43717080 | G | A | 0.822802 | -0.01433 | 0.002393 | 2.1E-09 | -0.012 | 0.013 | 0.36 | 0.000225 | 0.000225 |
| rs55909997 | 1 | 1.08E+08 | G | A | 0.772436 | -0.01355 | 0.00227 | 2.4E-09 | 0.0084 | 0.013 | 0.51 | 0.000224 | 0.000224 |
| rs56103247 | 20 | 62483184 | C | T | 0.967949 | 0.029765 | 0.004752 | 3.8E-10 | -0.05 | 0.031 | 0.1 | 0.000246 | 0.000246 |
| rs56291505 | 2 | 53891958 | A | G | 0.613553 | -0.0135 | 0.002413 | 2.2E-08 | 0.023 | 0.013 | 0.086 | 0.000197 | 0.000197 |
| rs56858768 | 13 | 86511730 | G | A | 0.809066 | -0.01488 | 0.00237 | 3.4E-10 | -0.02 | 0.013 | 0.13 | 0.000248 | 0.000248 |
| rs57585211 | 5 | 1.07E+08 | T | G | 0.761447 | -0.01673 | 0.00285 | 4.3E-09 | -0.0064 | 0.017 | 0.7 | 0.000216 | 0.000216 |
| rs6131281 | 20 | 11891724 | C | T | 0.693223 | 0.016081 | 0.002206 | 3.1E-13 | 0.03 | 0.012 | 0.016 | 0.000334 | 0.000334 |
| rs6141814 | 20 | 31368960 | C | A | 0.537546 | -0.0135 | 0.002226 | 1.3E-09 | -0.017 | 0.013 | 0.17 | 0.000231 | 0.000231 |
| rs62151809 | 2 | 1.04E+08 | C | T | 0.768315 | -0.01568 | 0.002174 | 5.4E-13 | -0.012 | 0.012 | 0.3 | 0.000327 | 0.000327 |
| rs62379379 | 5 | 1.41E+08 | G | T | 0.925824 | -0.02602 | 0.004232 | 7.8E-10 | -0.028 | 0.023 | 0.22 | 0.000237 | 0.000237 |
| rs62641636 | 2 | 68425427 | A | G | 0.682234 | 0.014424 | 0.002339 | 7E-10 | 0.0067 | 0.013 | 0.61 | 0.000239 | 0.000239 |
| rs6472942 | 8 | 76827190 | T | C | 0.565018 | -0.01316 | 0.002188 | 1.8E-09 | 0.024 | 0.012 | 0.053 | 0.000227 | 0.000227 |
| rs6551301 | 3 | 88294647 | T | C | 0.646978 | -0.01444 | 0.00239 | 1.5E-09 | 0.0046 | 0.014 | 0.74 | 0.000229 | 0.000229 |
| rs6673341 | 1 | 1.85E+08 | T | G | 0.700092 | -0.01451 | 0.002169 | 2.2E-11 | -0.017 | 0.012 | 0.16 | 0.000281 | 0.000281 |
| rs66852340 | 4 | 3311070 | C | T | 0.776099 | -0.01779 | 0.002601 | 7.9E-12 | -0.023 | 0.015 | 0.12 | 0.000294 | 0.000294 |
| rs6721975 | 2 | 5832667 | T | C | 0.81456 | -0.01669 | 0.002614 | 1.7E-10 | -0.0021 | 0.015 | 0.89 | 0.000256 | 0.000256 |
| rs6754968 | 2 | 1.05E+08 | G | A | 0.507784 | -0.01419 | 0.002265 | 3.7E-10 | -0.0053 | 0.013 | 0.67 | 0.000246 | 0.000246 |
| rs6797840 | 3 | 85656569 | A | C | 0.64881 | -0.0161 | 0.002184 | 1.7E-13 | -0.011 | 0.012 | 0.36 | 0.000341 | 0.000341 |
| rs6825241 | 4 | 1.53E+08 | C | A | 0.602106 | -0.01696 | 0.002166 | 4.9E-15 | -0.0056 | 0.012 | 0.64 | 0.000385 | 0.000385 |
| rs6850494 | 4 | 82291771 | A | C | 0.626832 | -0.01433 | 0.002222 | 1.1E-10 | -0.024 | 0.012 | 0.053 | 0.000261 | 0.000261 |
| rs6905544 | 6 | 98411631 | A | G | 0.652473 | -0.01899 | 0.00221 | 8.5E-18 | -0.023 | 0.012 | 0.062 | 0.000464 | 0.000464 |
| rs6973656 | 7 | 77422583 | A | G | 0.629579 | -0.01347 | 0.002206 | 1E-09 | 0.0053 | 0.012 | 0.66 | 0.000234 | 0.000234 |
| rs6996198 | 8 | 65463442 | C | T | 0.805403 | -0.0164 | 0.002974 | 3.5E-08 | -0.03 | 0.017 | 0.075 | 0.000191 | 0.000191 |
| rs7089973 | 10 | 1.17E+08 | C | A | 0.679945 | -0.01336 | 0.002237 | 2.4E-09 | 0.0072 | 0.013 | 0.57 | 0.000224 | 0.000224 |
| rs7157001 | 14 | 99749484 | A | G | 0.629579 | -0.01397 | 0.002498 | 2.3E-08 | -0.016 | 0.016 | 0.33 | 0.000196 | 0.000196 |
| rs7184800 | 16 | 53509131 | G | A | 0.760989 | 0.016817 | 0.002349 | 8.2E-13 | 0.013 | 0.013 | 0.33 | 0.000322 | 0.000322 |
| rs7189927 | 16 | 28913787 | T | C | 0.751832 | 0.014974 | 0.002259 | 3.4E-11 | 0.021 | 0.013 | 0.11 | 0.000276 | 0.000276 |
| rs7248205 | 19 | 10770305 | C | T | 0.51511 | 0.013925 | 0.002217 | 3.4E-10 | 0.016 | 0.013 | 0.21 | 0.000248 | 0.000248 |
| rs72723237 | 1 | 1.88E+08 | C | T | 0.988095 | -0.04663 | 0.007942 | 4.3E-09 | -0.047 | 0.045 | 0.3 | 0.000217 | 0.000217 |
| rs72781699 | 2 | 24277709 | G | A | 0.842949 | -0.01868 | 0.002678 | 3E-12 | -0.0066 | 0.015 | 0.66 | 0.000306 | 0.000306 |
| rs72828890 | 5 | 1.68E+08 | C | T | 0.949634 | 0.019292 | 0.003298 | 4.9E-09 | 0.018 | 0.02 | 0.35 | 0.000215 | 0.000215 |
| rs72834698 | 6 | 26176517 | G | A | 0.935897 | 0.022656 | 0.003101 | 2.7E-13 | 0.027 | 0.018 | 0.14 | 0.000335 | 0.000335 |
| rs749671 | 16 | 31088347 | G | A | 0.537088 | 0.015654 | 0.002238 | 2.7E-12 | 0.012 | 0.013 | 0.33 | 0.000307 | 0.000307 |
| rs7564130 | 2 | 50606642 | T | C | 0.584707 | -0.01499 | 0.002251 | 2.8E-11 | 0.002 | 0.013 | 0.87 | 0.000278 | 0.000278 |
| rs7693703 | 4 | 1.18E+08 | G | A | 0.940476 | 0.022844 | 0.003841 | 2.7E-09 | 0.0063 | 0.021 | 0.76 | 0.000222 | 0.000222 |
| rs7700107 | 4 | 17880416 | A | C | 0.857601 | -0.02106 | 0.003138 | 1.9E-11 | -0.044 | 0.019 | 0.018 | 0.000283 | 0.000283 |
| rs7716447 | 5 | 88800331 | A | G | 0.791667 | -0.01338 | 0.002269 | 3.6E-09 | 0.025 | 0.016 | 0.13 | 0.000219 | 0.000219 |
| rs7865801 | 9 | 14745886 | G | A | 0.598901 | 0.012745 | 0.002262 | 1.8E-08 | 0.027 | 0.013 | 0.034 | 0.000199 | 0.000199 |
| rs801733 | 11 | 65934549 | A | C | 0.77152 | 0.016858 | 0.002253 | 7.3E-14 | 0.033 | 0.013 | 0.0097 | 0.000351 | 0.000351 |
| rs8043253 | 15 | 41503730 | C | T | 0.554487 | -0.01207 | 0.002187 | 3.4E-08 | -0.002 | 0.012 | 0.87 | 0.000191 | 0.000191 |
| rs8756 | 12 | 66359752 | C | A | 0.672161 | -0.01345 | 0.002166 | 5.3E-10 | -0.045 | 0.012 | 0.00019 | 0.000242 | 0.000242 |
| rs9471333 | 6 | 40362023 | C | T | 0.527015 | 0.013104 | 0.002169 | 1.5E-09 | 0.028 | 0.012 | 0.019 | 0.000229 | 0.000229 |
| rs9563168 | 13 | 54247827 | G | A | 0.839286 | 0.017587 | 0.002667 | 4.3E-11 | -0.015 | 0.015 | 0.29 | 0.000273 | 0.000273 |
| rs9569734 | 13 | 58319476 | A | G | 0.782509 | 0.018885 | 0.003007 | 3.4E-10 | 0.042 | 0.017 | 0.012 | 0.000248 | 0.000248 |
| rs9718104 | 6 | 1.66E+08 | T | G | 0.911172 | -0.04077 | 0.00461 | 9.3E-19 | -0.01 | 0.028 | 0.71 | 0.000491 | 0.000491 |
| rs973734 | 7 | 89387578 | C | A | 0.924451 | 0.017076 | 0.003013 | 1.5E-08 | 0.028 | 0.017 | 0.093 | 0.000202 | 0.000202 |
| rs9834970 | 3 | 36856030 | T | C | 0.506868 | 0.012768 | 0.002158 | 3.3E-09 | 0.029 | 0.012 | 0.021 | 0.00022 | 0.00022 |
| rs984409 | 1 | 67020440 | G | A | 0.794872 | -0.01487 | 0.002253 | 4.1E-11 | -0.025 | 0.013 | 0.046 | 0.000273 | 0.000273 |
| rs9867121 | 3 | 1.15E+08 | C | A | 0.70467 | 0.019499 | 0.002809 | 3.9E-12 | -0.0007 | 0.016 | 0.96 | 0.000303 | 0.000303 |
| rs9902312 | 17 | 65070304 | T | C | 0.538462 | 0.015324 | 0.002327 | 4.5E-11 | -0.016 | 0.013 | 0.21 | 0.000272 | 0.000272 |
| rs9964724 | 18 | 35159124 | C | T | 0.669872 | 0.017646 | 0.002327 | 3.3E-14 | 0.0082 | 0.013 | 0.53 | 0.000361 | 0.000361 |

## Table S4. List of instrucment variables used from the breakfast skipping to T2D

| SNP | CHR | BP | A1 | A2 | EAF | breakfast skipping | | | T2D | | | r2 |
| --- | --- | --- | --- | --- | --- | --- | --- | --- | --- | --- | --- | --- |
|  |  |  |  |  |  | BETA | SE | P | BETA | SE | P |  |
| rs35107470 | 15 | 74817689 | G | A | 0.871337 | 0.018514 | 0.002953 | 3.5E-10 | 0.011 | 0.015 | 0.45 | 0.000247 |
| rs6017427 | 20 | 43467380 | A | G | 0.845238 | 0.021385 | 0.003856 | 3.1E-08 | -0.011 | 0.018 | 0.54 | 0.000193 |
| rs637174 | 19 | 49266936 | A | G | 0.555861 | 0.016087 | 0.002863 | 1.8E-08 | 0.013 | 0.018 | 0.49 | 0.000198 |
| rs6986473 | 8 | 64487672 | T | C | 0.753205 | 0.017478 | 0.003211 | 4.2E-08 | -0.028 | 0.015 | 0.072 | 0.000186 |
| rs8097544 | 18 | 1839564 | G | A | 0.840201 | 0.032552 | 0.003821 | 1.8E-17 | 0.03 | 0.017 | 0.08 | 0.000456 |

## Table S5. List of SNPs with genome-wide significance for potential confounding traits in TV watching IVs searched from GWAS catelog

| SNP | Confounder |
| --- | --- |
| rs10189857 | self reported educational attainment |
| rs10890123 | self reported educational attainment |
| rs10994943 | smoking behavior |
| rs11657730 | primary hypertension |
| rs12554512 | body weight |
| rs13107325 | body mass index, physical activity, hypertension, smoking behavior, alcohol consumption, body fat percentage |
| rs17789218 | Waist-hip index |
| rs303753 | alcohol consumption, diet measurement |
| rs4775373 | Cardiovascular disease |
| rs62379379 | Cognitive performance |
| rs6754968 | Body mass index |
| rs6973656 | Body mass index |
| rs72834698 | Educational attainment, Waist circumference adjusted for body mass index |
| rs9471333 | Body fat percentage |
| rs9563168 | self reported educational attainment |
| rs9964724 | self reported educational attainment |

# Supplementary Figure

## Figure S1: Leave-one-out plot for univariable analysis of TV watching on T2D


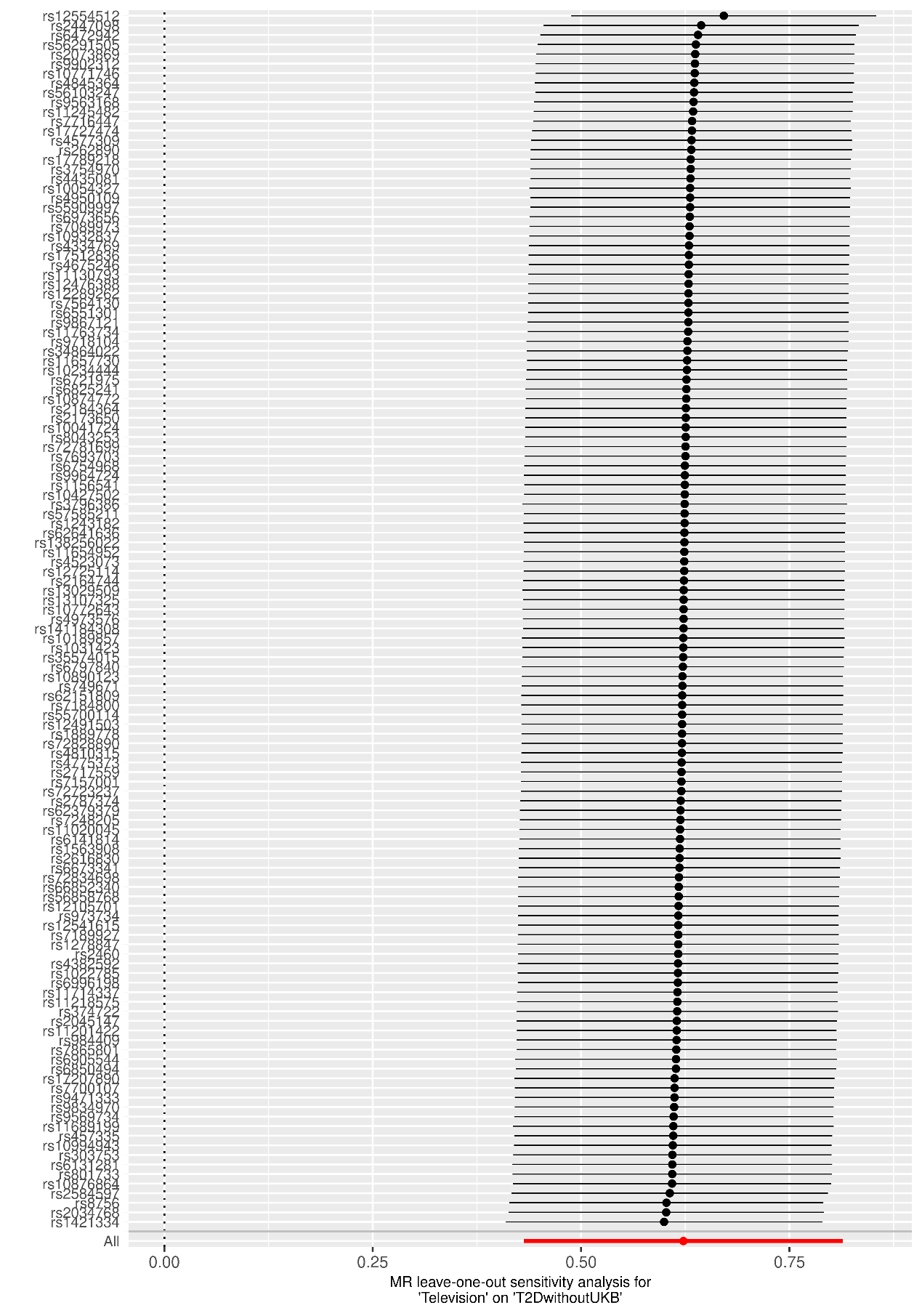


## Figure S2: Leave-one-out plot for univariable analysis of breakfast skipping on T2D


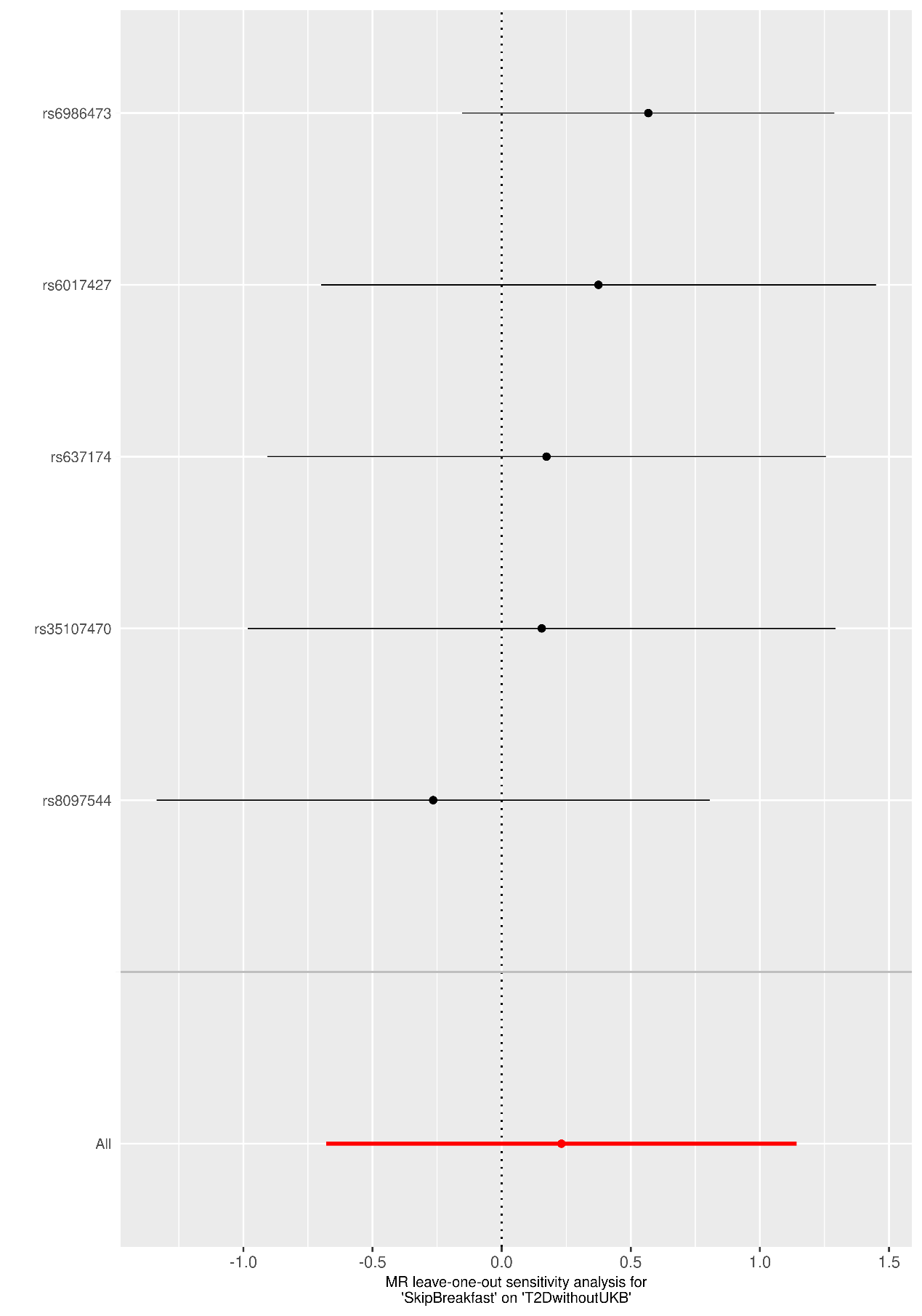


## Figure S3: Leave-one-out plot for univariable analysis of T2D on TV watching


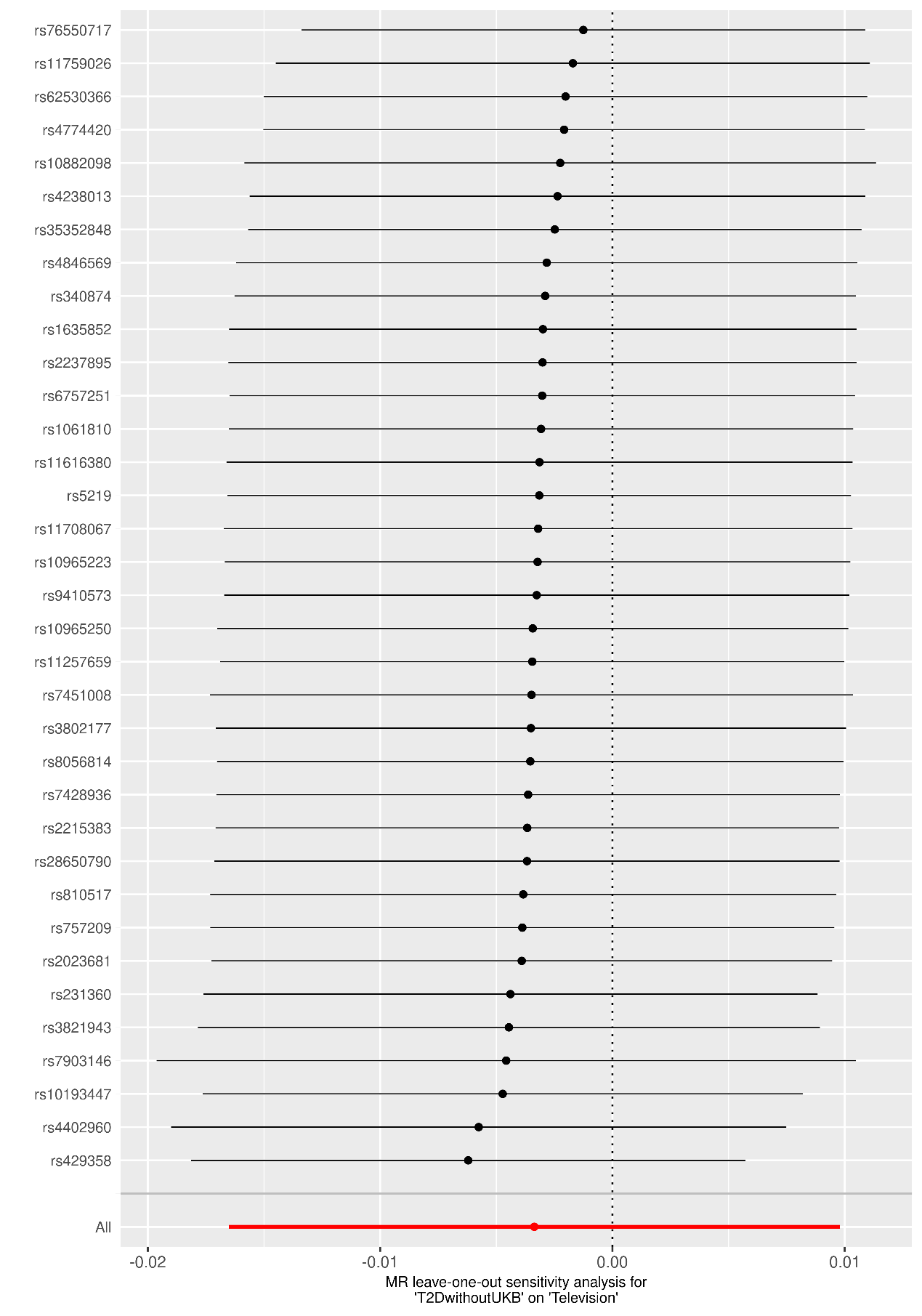


## Figure S4: Leave-one-out plot for univariable analysis of T2D on breakfast skipping


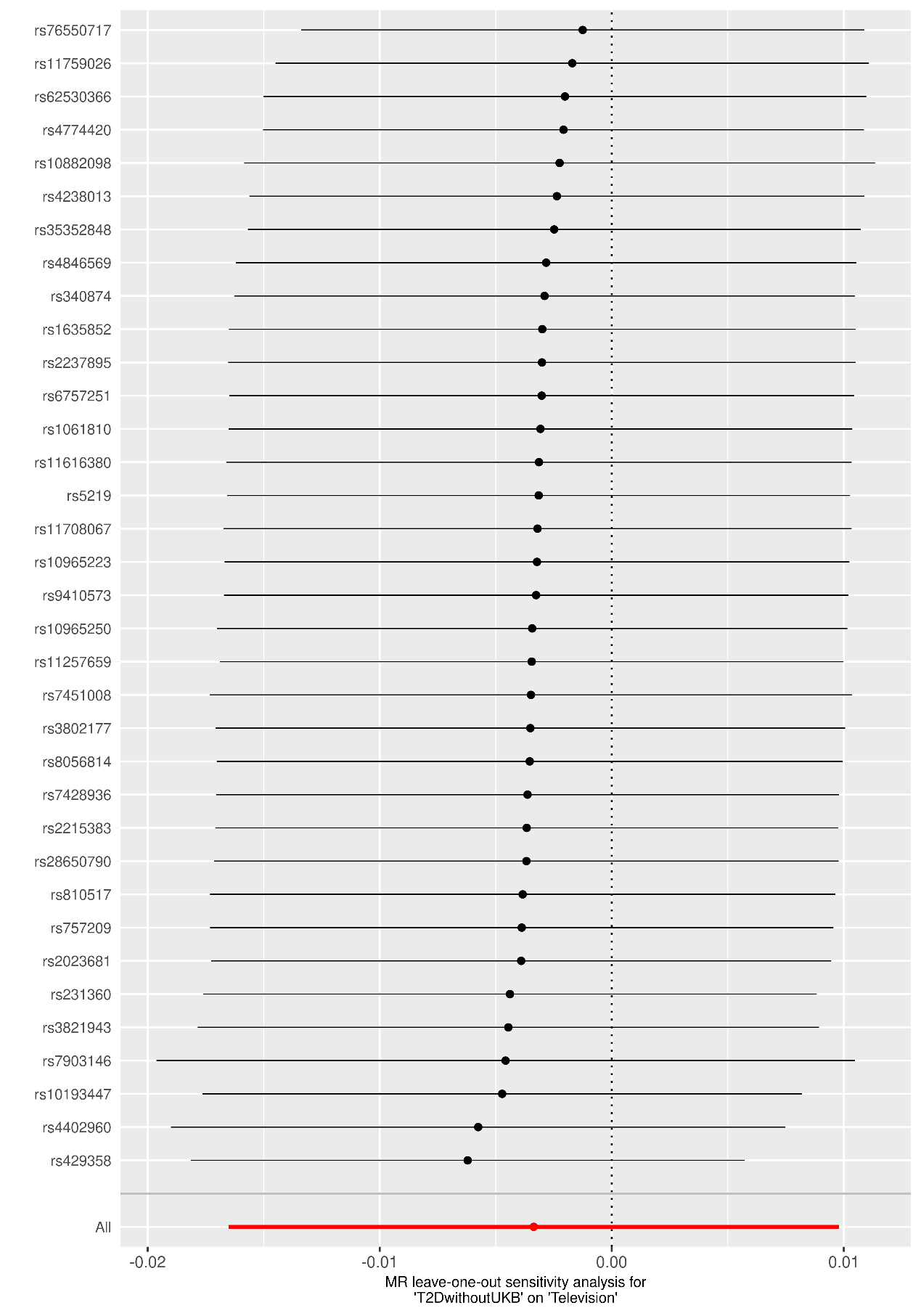


## Figure S5: Funnel plot assessing the extent to which pleiotropy is balanced across the set of instruments used in the univariable MR analysis of TV watching on T2D


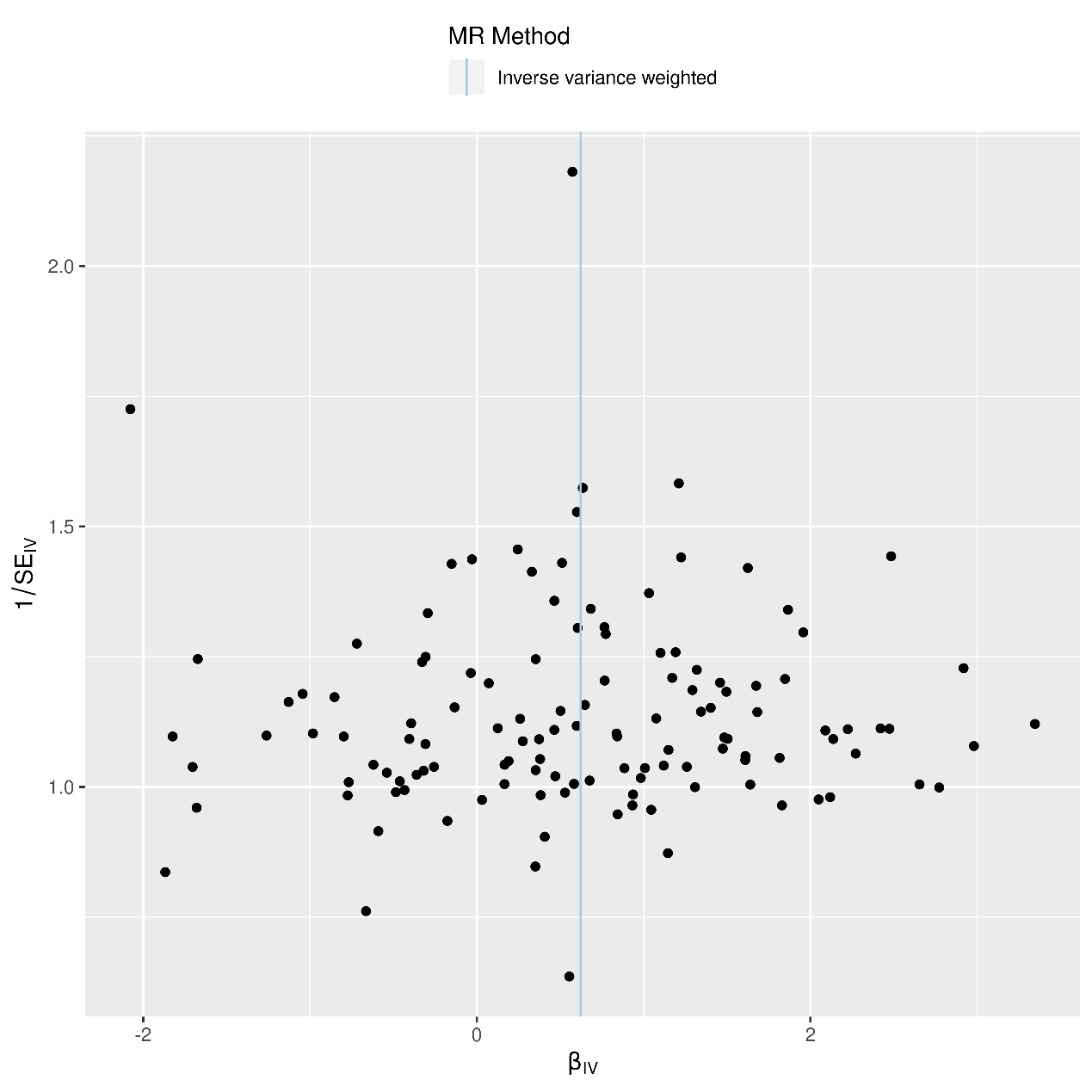


## Figure S6: Funnel plot assessing the extent to which pleiotropy is balanced across the set of instruments used in the univariable MR analysis of breakfast skipping on T2D


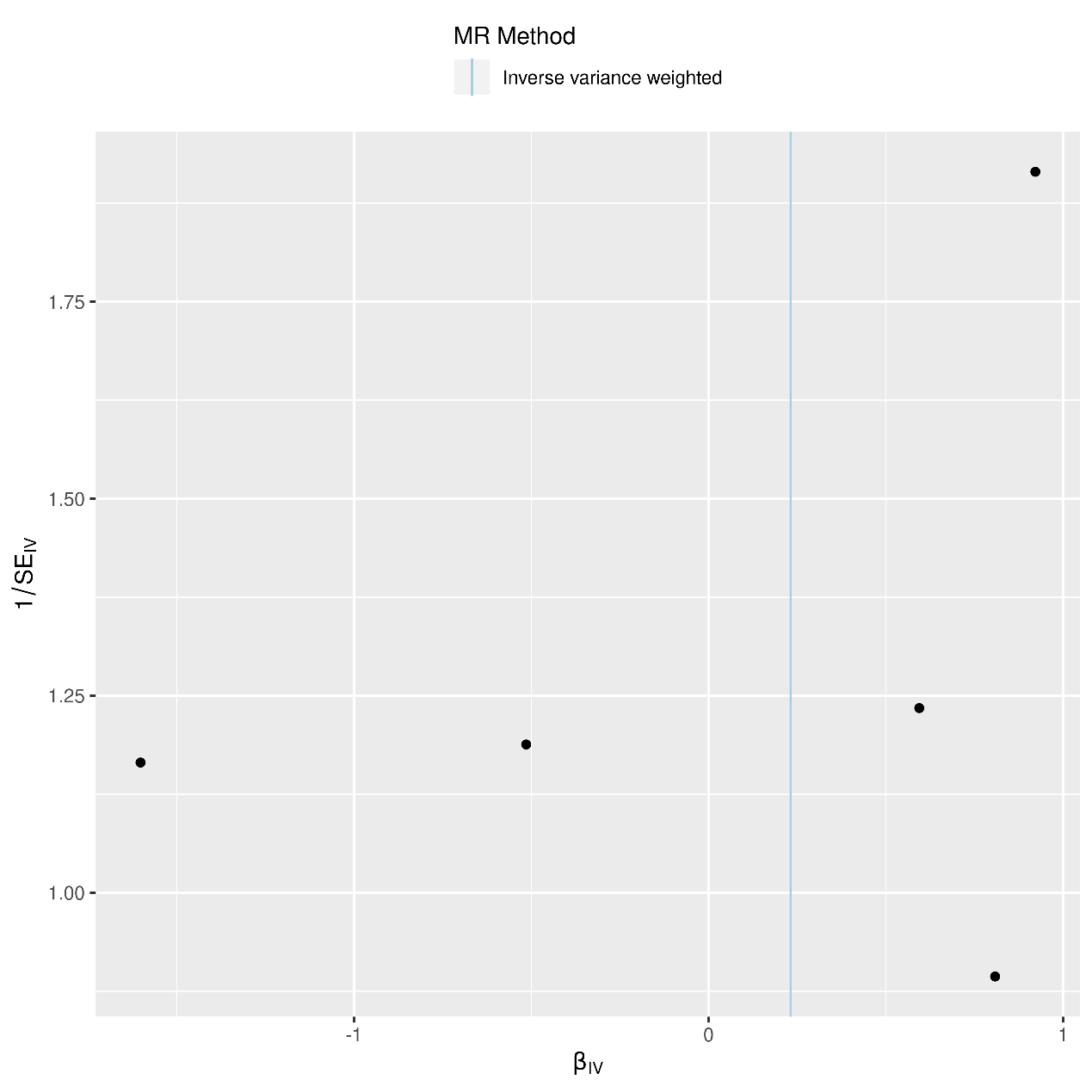


## Figure S7: Funnel plot assessing the extent to which pleiotropy is balanced across the set of instruments used in the univariable MR analysis of T2D on TV watching


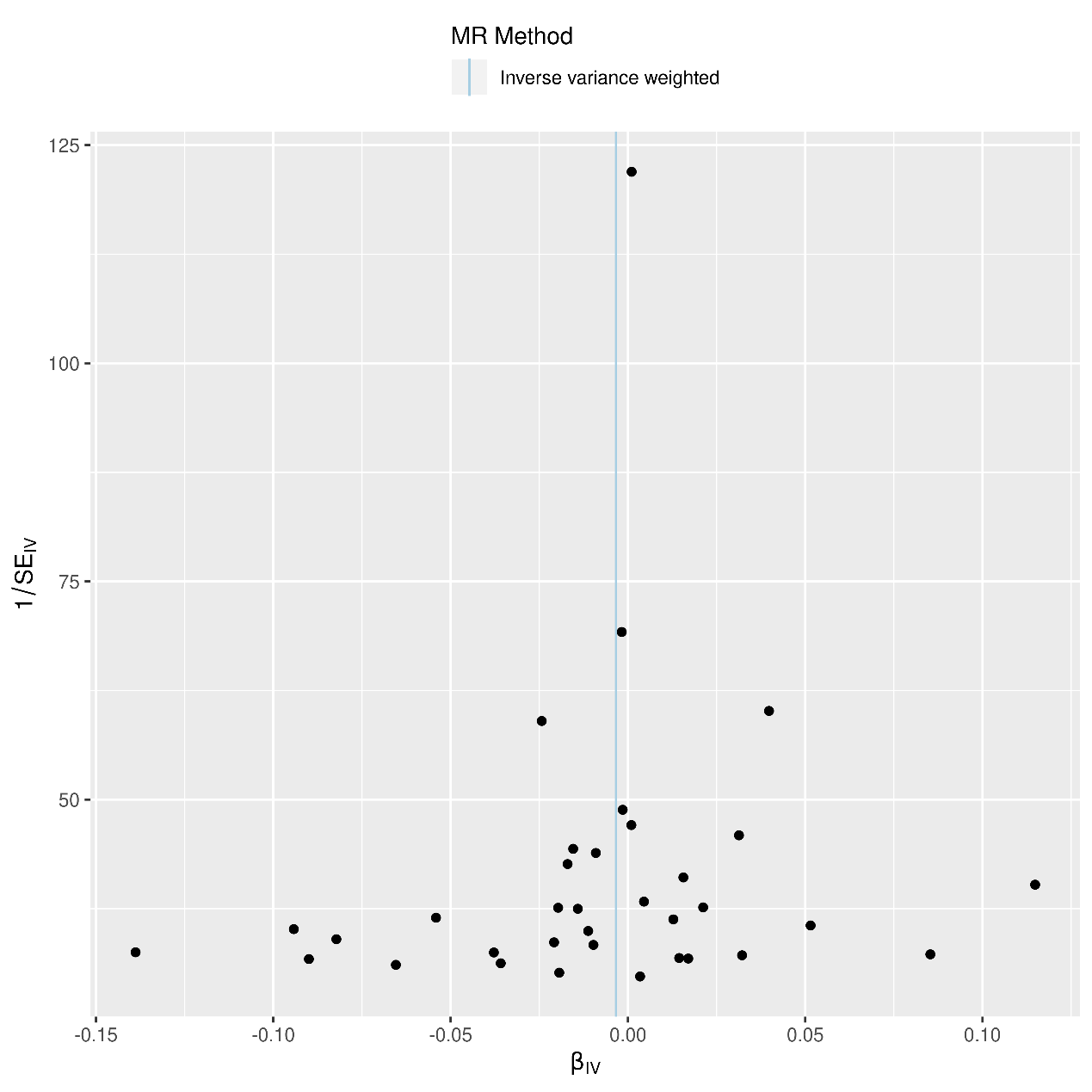


## Figure S8: Funnel plot assessing the extent to which pleiotropy is balanced across the set of instruments used in the univariable MR analysis of T2D on breakfast skipping


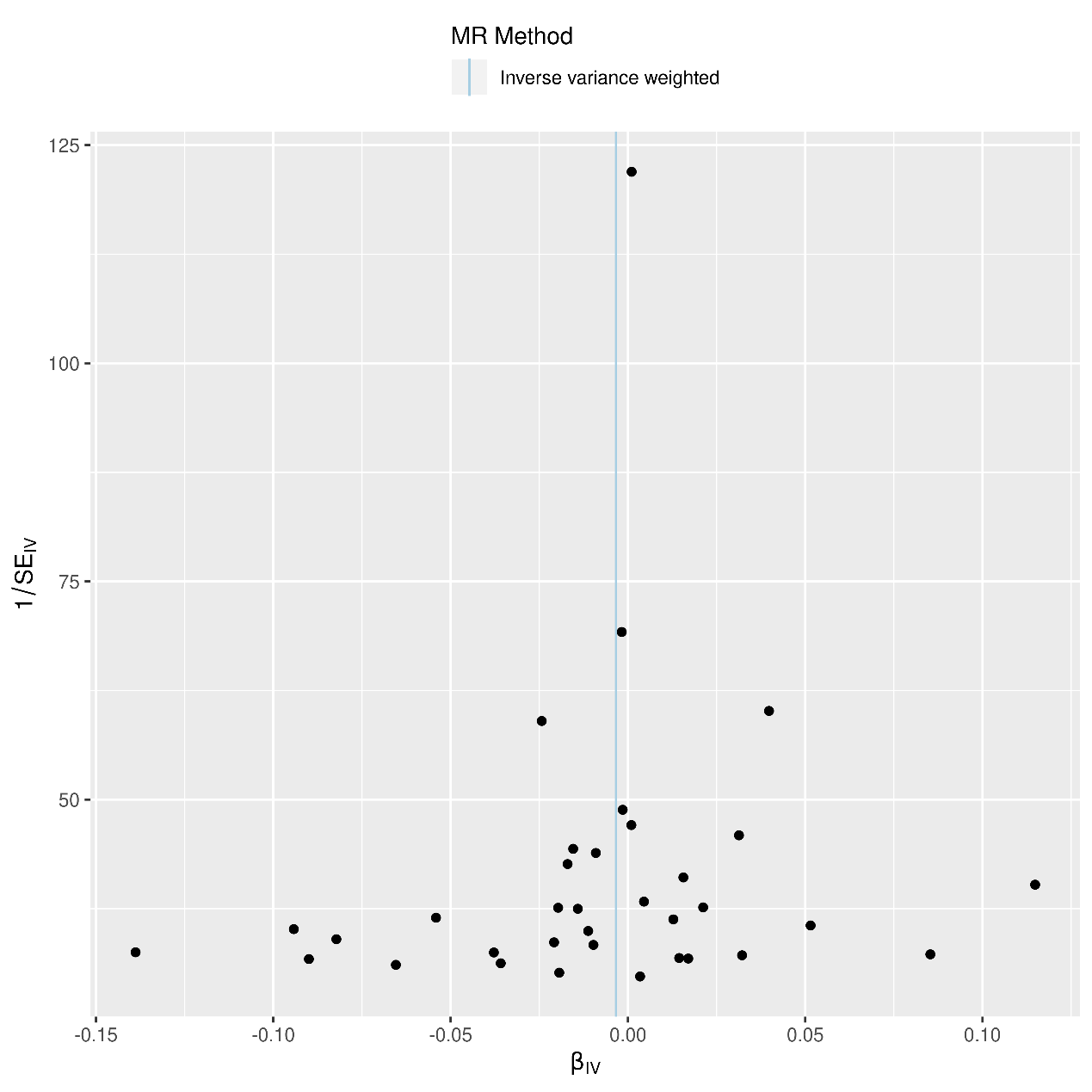

Supplement: Supplementary file 1 [file DataSheet_1.docx]
